# Deletion of BDNF in Pax2 Lineage-Derived Interneuron Precursors in the Hindbrain Hampers the Proportion of Excitation/Inhibition, Learning, and Behavior

**DOI:** 10.3389/fnmol.2021.642679

**Published:** 2021-03-26

**Authors:** Philipp Eckert, Philine Marchetta, Marie K. Manthey, Michael H. Walter, Sasa Jovanovic, Daria Savitska, Wibke Singer, Michele H. Jacob, Lukas Rüttiger, Thomas Schimmang, Ivan Milenkovic, Peter K. D. Pilz, Marlies Knipper

**Affiliations:** ^1^Department of Otolaryngology, Head and Neck Surgery, Tübingen Hearing Research Centre, Molecular Physiology of Hearing, University of Tübingen, Tübingen, Germany; ^2^Department of Neuroscience, Sackler School of Biomedical Sciences, Tufts University School of Medicine, Boston, MA, United States; ^3^Department for Animal Physiology, Institute of Neurobiology, University of Tübingen, Tübingen, Germany; ^4^School of Medicine and Health Sciences, Carl von Ossietzky University Oldenburg, Oldenburg, Germany; ^5^Instituto de Biología y Genética Molecular, Consejo Superior de Investigaciones Científicas, Universidad de Valladolid, Valladolid, Spain

**Keywords:** BDNF, Pax2, Arc/Arg3.1, GABAergic interneuron, parvalbumin interneuron, autism spectrum disorder

## Abstract

Numerous studies indicate that deficits in the proper integration or migration of specific GABAergic precursor cells from the subpallium to the cortex can lead to severe cognitive dysfunctions and neurodevelopmental pathogenesis linked to intellectual disabilities. A different set of GABAergic precursors cells that express Pax2 migrate to hindbrain regions, targeting, for example auditory or somatosensory brainstem regions. We demonstrate that the absence of BDNF in Pax2-lineage descendants of *Bdnf^*Pax*2^*KOs causes severe cognitive disabilities. In *Bdnf^*Pax*2^*KOs, a normal number of parvalbumin-positive interneurons (**PV-IN**s) was found in the auditory cortex (**AC**) and hippocampal regions, which went hand in hand with reduced PV-labeling in neuropil domains and elevated activity-regulated cytoskeleton-associated protein (**Arc/Arg3.1**; here: **Arc**) levels in pyramidal neurons in these same regions. This immaturity in the inhibitory/excitatory balance of the AC and hippocampus was accompanied by elevated LTP, reduced (sound-induced) LTP/LTD adjustment, impaired learning, elevated anxiety, and deficits in social behavior, overall representing an autistic-like phenotype. Reduced tonic inhibitory strength and elevated spontaneous firing rates in dorsal cochlear nucleus (**DCN**) brainstem neurons in otherwise nearly normal hearing *Bdnf^*Pax*2^*KOs suggests that diminished fine-grained auditory-specific brainstem activity has hampered activity-driven integration of inhibitory networks of the AC in functional (hippocampal) circuits. This leads to an inability to scale hippocampal post-synapses during LTP/LTD plasticity. BDNF in Pax2-lineage descendants in lower brain regions should thus be considered as a novel candidate for contributing to the development of brain disorders, including autism.

## Introduction

Current views suggest that γ-aminobutyric acid (**GABA**) interneurons (**INs**) that populate the cerebral cortex, striatum, hippocampus, or olfactory bulb, mainly derive from progenitor cells in the subpallium, from where they migrate in multiple streams to reach their destinations (Guo and Anton, 2014). Numerous studies suggest that dysfunctions of cortical GABAergic INs are linked with various neurodevelopmental disorders (Marin, 2012; Gao and Penzes, 2015; Uehara et al., 2015; Canetta et al., 2016; Ferguson and Gao, 2018; Lim et al., 2018; Skene et al., 2018; Lunden et al., 2019; Miyoshi, 2019; Wei et al., 2020).

Whereas the intrinsic migratory fate of subpallium-derived GABAergic IN precursors is characterized by the expression of the transcription factors Pax6 (Guo and Anton, 2014), Pax2-expressing (**Pax2+)** GABAergic IN precursors migrate from the ventricular zones to lower brain levels, such as the cerebellum (**Cb**), hindbrain and spinal cord, which are posterior to midbrain regions (Nornes et al., 1990; Maricich and Herrup, 1999; Rowitch et al., 1999; Larsson, 2017), and to a few thalamic frontal brain regions (Maricich and Herrup, 1999; Fotaki et al., 2008).

The role of Pax2-lineage descendants in brain function, particularly for higher brain function or neurodevelopmental disorders, is elusive. We have previously observed that a deletion of brain-derived nerve growth factor (**BDNF**) under the Pax2 promoter in *Bdnf^*Pax*2^*KOs leads to circling behavior and does not profoundly alter basal auditory function, but diminishes fast auditory processing (Zuccotti et al., 2012; Chumak et al., 2016).

With sensory experience, fast auditory processing matures after hearing onset by the maturation of improved receptive fields following integration of inhibitory cortical networks into functional fronto-striatal circuits (Xu et al., 2010). Proper integration of inhibitory networks in functional fronto-striatal circuits is a predicted prerequisite for improved auditory perception and memory-dependent signal amplification processes (Kraus and White-Schwoch, 2015; Weinberger, 2015; Irvine, 2018; Knipper et al., 2020). Fast auditory processing is also suggested to be essential for memory-dependent central auditory adjustment processes following enriching sound exposure (**SE**) or auditory deprivation (Matt et al., 2018; Knipper et al., 2020). Accordingly, long-term plasticity changes following SE or auditory deprivation can be monitored through altered levels of activity-regulated cytoskeleton-associated protein (**Arc/Arg3.1**; here: **Arc**) and parvalbumin (**PV**) in the AC and hippocampus and through correlating changes in long-term potentiation (**LTP**) in hippocampal CA1 pyramidal neurons (Matt et al., 2018; Marchetta et al., 2020). Therefore, sound-induced adjustment processes are likely to be reflected by altered plasticity changes in the AC and hippocampus.

We here demonstrate that BDNF deletion in Pax2-lineage descendants in hindbrain regions of *Bdnf^*Pax*2^*KO mice leads to elevated thresholds, lower dynamic range and diminished inhibitory strength of auditory brainstem responses in the DCN. *Bdnf^*Pax*2^*KO mice moreover exhibit reduced PV-IN and elevated Arc labeling in the AC and hippocampus, suggesting that diminished auditory brainstem output activity has hampered activity-dependent integration of cortical GABAergic INs into functional hippocampal circuits. Accordingly, *Bdnf^*Pax*2^*KOs developed elevated hippocampal LTP, deficits in LTP and long-term depression (**LTD**) adjustment to SE, and deficits in learning, social behavior, or anxiety control, altogether resembling an autistic-like phenotype. The role of BDNF in Pax2-lineage descendants in lower hindbrain regions thus needs to be revisited in the context of neurodevelopmental disorders, such as autism spectrum disorder (**ASD**).

## Results

### BDNF Is Present in Pax2-Lineage Descendants in Brainstem and Hypothalamic Regions but Not in Cortical and Hippocampal Regions

**FIGURE 1:**
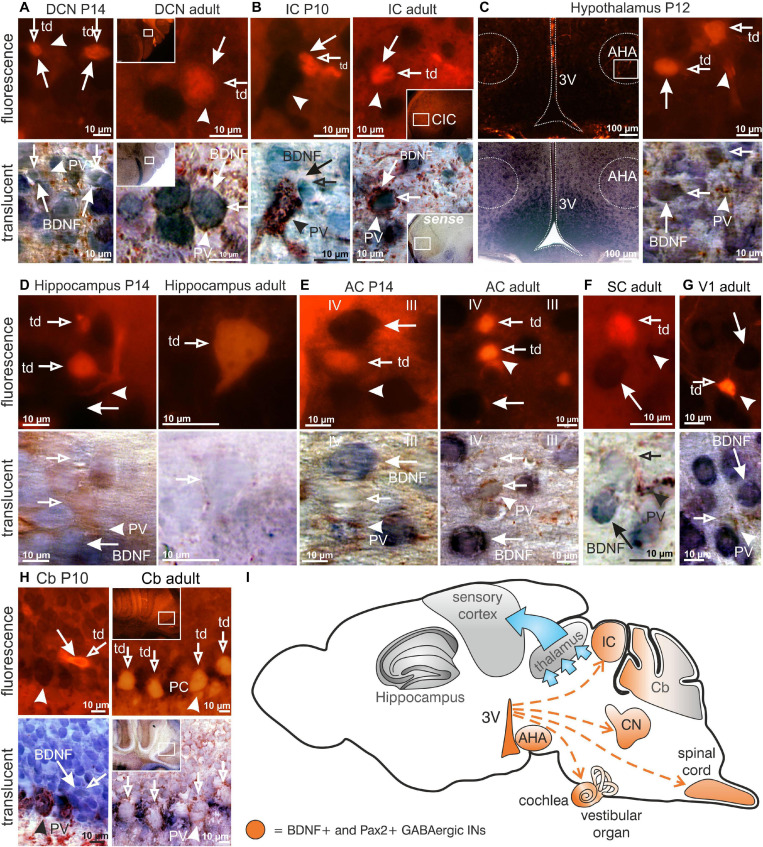
Brain-derived nerve growth factor expression in Pax2-CRE-Rosa^tdTomato^ reporter mice. **(A)** In P14 and adult Pax2-CRE-Rosa^tdTomato^ mice Pax2-Cre (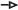) in the DCN, **(B)** as well as in the P10 and adult IC was co-localized with BDNF mRNA (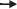) and PV (▶). **(C)** In Pax2-CRE-Rosa^tdTomato^ reporter mice at the age of P12, Pax2 (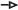) in the anterior hypothalamic area (APA) was co-localized with BDNF mRNA (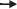), as well as with PV (▶). **(D,E)** Pax2-CRE-Rosa^tdTomato^ reporter mice showed neither co-localization of Pax2+ cells (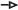) with BDNF mRNA (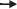) in between layer III and IV in the AC at the age of P14, nor did adult animals. They also did not show co-localization of Pax2+ cells (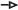) with BDNF mRNA (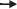) in the CA1 region at the age of P14 or in adult animals. **(F)** Adult Pax2-CRE-Rosa^tdTomato^ reporter mice showed no co-localization of Pax2-Cre (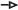) with BDNF mRNA (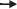) in the superior colliculus, **(G)** visual cortex, **(H)**, as well as in the adult Cb. The Purkinje cells were surrounded by PV (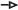). At P10, some fusiform-shaped Pax2+ cells (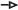) overlapped with BDNF mRNA (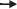). **(I)** Scheme of BDNF+ Pax2-lineage descendants migration (red dashed arrows) to the cochlea, DCN, IC and AHA starting in the third ventricle (3V). Scale bar = 10 μM with the exception of **(E)** left panel, where it is 100 μm.

The use of Rosa^tdTomato^ reporter mice (Madisen et al., 2010) crossed with Pax2-Cre mice, here called Pax2-CRE-Rosa^tdTomato^ mice, allowed us to efficiently visualize Cre-directed gene expression through native red fluorescence simultaneously with BDNF mRNA. We combined labeling for BDNF mRNA and PV-protein with tdTomato fluorescence to specify presumptive BDNF-expressing (**BDNF+)** Pax2-Cre descendent neurons during the maturation process of PV-INs throughout the critical developmental time period after hearing onset between P10-P14. This is the time period when cortical PV-IN networks are integrated in functional fronto-striatal circuits after tangential migrating GABAergic INs have reached their final destination (Kimura and Itami, 2019). We found that BDNF was expressed in Pax2-lineage descendants during the critical developmental time period and in adults in neuronal cells of the auditory brainstem but not in frontal brain regions. Representative images are shown for distinct regions between P10 and P14 or at the adult stage, such as the DCN (Figure 1A, left panel P14; right panel adult), inferior colliculus (**IC**; Figure 1B, left panel P10; right panel adult) and the anterior hypothalamic area (**AHA**; Figure 1C, P12). No labeling was observed in thalamic and frontal regions such as the hippocampus (Figure 1D, left panel P14; right panel adult), the AC (Figure 1E, left panel P14; right panel adult), superior colliculus (**SC**; Figure 1F, adult), visual cortex (**V1**; Figure 1G, adult), or somatosensory cortex (not shown). In the Cb an overlap of BDNF mRNA and tdTomato expression was only seen at P10 but not beyond this stage (Figure 1H, left panel P10; right panel adult).

**In conclusion,** BDNF is found in Pax2-lineage descendants in lower brainstem regions and AHA, but not in the thalamus, Cb, or frontal cortical regions including the hippocampus, visual and somatosensory systems.

### *Bdnf^*Pax*2^*KOs Exhibit Reduced PV-IN Labeling in Neuropil Regions and Elevated Levels of Arc in the AC and Hippocampus

Given that *Bdnf^*Pax*2^*KO mice show diminished auditory processing (Zuccotti et al., 2012; Chumak et al., 2016) which has been shown to influence plasticity genes and LTP in hippocampal circuits (Matt et al., 2018), we considered an impact of BDNF+ Pax2-lineage descendants in lower brainstem regions on higher brain functions. To test this hypothesis we analyzed PV immunostaining as a marker for fast-spiking, GABAergic INs (Cardin et al., 2009; Hu et al., 2014; Kim et al., 2016). As a marker for excitatory neurons, we analyzed the expression of Arc, which is preferentially expressed in these neurons (Tzingounis and Nicoll, 2006; Bramham et al., 2008).

**FIGURE 2:**
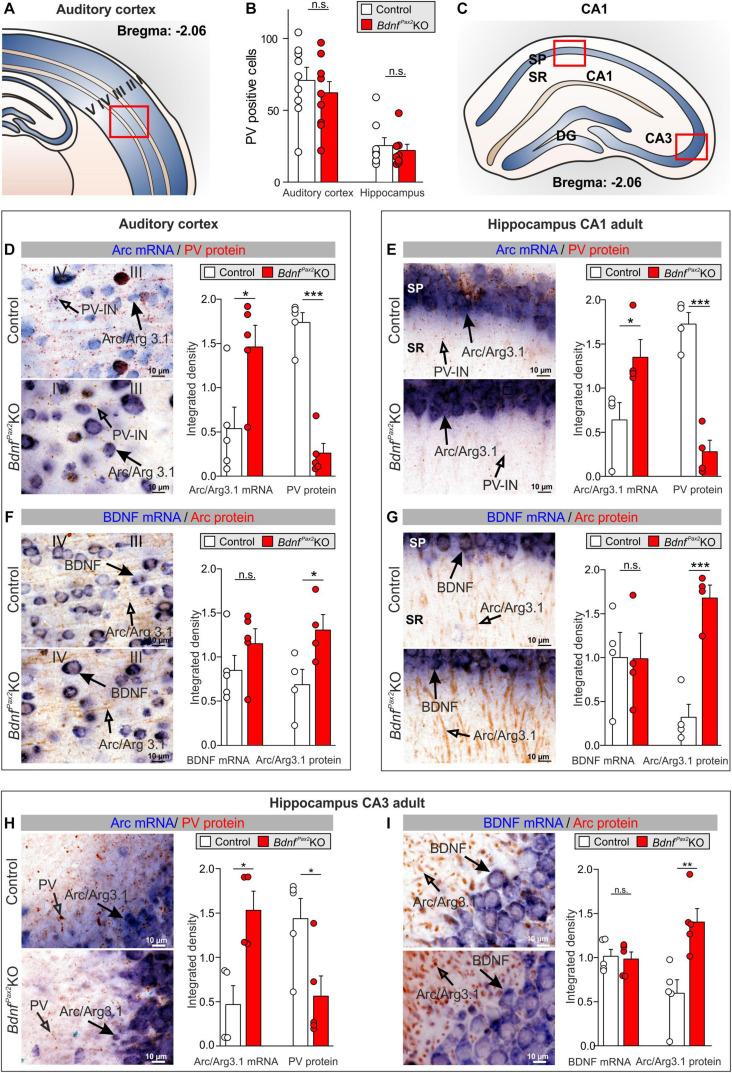
Parvalbumin immunostaining and Arc/BDNF expression in *Bdnf^*Pax*2^*KOs. **(A)** Scheme of the AC with layers I to V. **(B)** No difference in number of PV+ cells in the AC and hippocampus between *Bdnf^*Pax*2^*KOs and controls (*n* = 8 each; *P* > 0.05). **(C)** Scheme of the hippocampus with CA1 and CA3 region and DG. **(D)** *Bdnf^*Pax*2^*KOs showed increased Arc mRNA (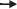, *n* = 5 each; *P* = 0.028) and decreased PV protein levels (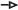, *n* = 5 each; *P* < 0.0001) in the AC in comparison to controls, as well as **(E)** increased Arc mRNA (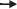, *n* = 4 each; *P* = 0.042) and decreased perisomatic and dendritic PV protein levels (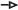, *n* = 4 each; *P* < 0.001) in the hippocampal CA1 region. **(F)** *Bdnf^*Pax*2^*KOs revealed similar levels of BDNF mRNA in the AC (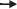, *n* = 5 each; *P* = 0.243), but increased Arc protein (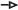, *n* = 4 each; *P* = 0.0453) compared to controls. **(G)** BDNF mRNA (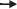, *n* = 4 each; *P* = 0.979) remained unaltered in *Bdnf^*Pax*2^*KOs, while Arc protein (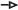, *n* = 4 each; *P* < 0.001) was increased in hippocampal CA1 regions in comparison to controls. Scale bar **(C–F)** = 10 μm, **(G)** = 100 μm. Mean ± S.E.M. **(H)** *Bdnf^*Pax*2^*KOs showed increased Arc mRNA (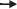, *n* = 4 each; *P* = 0.013) and decreased perisomatic and dendritic PV protein levels (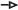, *n* = 4 each; *P* = 0.026) in the hippocampal CA3 region in comparison to controls. **(I)** BDNF mRNA (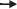, *n* = 4 each; *P* = 0.90) remained unaltered in *Bdnf^*Pax*2^*KOs, while Arc protein (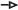, *n* = 4 each; *P* = 0.006) was increased in hippocampal CA3 regions, in comparison to controls. n.s. = *P* > 0.05, * = *P* < 0.05, ** = *P* < 0.01, *** = P < 0.001.

Neuronal cell counts of PV-INs in *Bdnf^*Pax*2^*KO mice revealed no differences compared to controls as shown for the AC in layer III/IV [Figures 2A,B; unpaired two-tailed student’s *t*-test, *t*(14) = 0.4877, *P* = 0.6333, *n* = 8 mice each] and the hippocampus [Figures 2B,C; unpaired two-tailed student’s *t*-test, *t*(14) = 0.7959, *P* = 0.4377, *n* = 8 mice each]. This observation indicates that in *Bdnf^*Pax*2^*KO mice, GABAergic INs of cortical and hippocampal regions have successfully reached their destination.

Next, we focused on PV-IN labeling in neuropil domains which are enriched in dendritic synapses and filopodia as for instance in layer III/IV of the AC, or the stratum radiatum and stratum lucidum of hippocampal CA1/CA3 regions, respectively. Quantification of PV-IN, and Arc mRNA levels revealed a strikingly reduced staining of PV-IN, parallel to the elevated Arc mRNA levels in *Bdnf^*Pax*2^*KOs, as compared to controls (Figure 2). This was shown for layer III/IV in the AC [Figures 2A,D; unpaired two-tailed student’s *t*-test, PV: *t*(8) = 9.482, *P* < 0.0001, Arc: *t*(8) = 2.686, *P* = 0.0277, *n* = 5 mice each] and for the hippocampus in the stratum pyramidale and stratum radiatum of the CA1 region [Figures 2C,E; unpaired two-tailed student’s *t*-test, PV: *t*(6) = 7.816, *P* = 0.0002, Arc: *t*(6) = 2.586, *P* = 0.0415, *n* = 4 mice each] as well as for the CA3 region [Figure 2H; unpaired two-tailed student’s *t*-test, PV: *t*(8) = 2.718, *P* = 0.0263, Arc: *t*(6) = 3.501, *P* = 0.0128, *n* = 4 mice each]. The levels of BDNF mRNA were not different between *Bdnf^*Pax*2^*KOs and controls [Figures 2F,G,I; unpaired two-tailed student’s *t*-test, AC: *t*(8) = 1.262, *P* = 0.2426, *n* = 5 mice each, CA1: *t*(6) = 0.07922, *P* = 0.9394, *n* = 4 mice each, CA3: *t*(8) = 0.2594, *P* = 0.8019, *n* = 4 mice each]. In the same sections in which no difference of BDNF mRNA levels was observed, elevated levels of Arc protein were visible; this was shown for the AC [Figure 2F; unpaired two-tailed student’s *t*-test, *t*(6) = 2.519, *P* = 0.0453], and for the hippocampus in CA1 [Figure 2G; unpaired two-tailed student’s *t*-test, *t*(6) = 6.526, *P* = 0.0006] and CA3 regions [Figure 2I; unpaired two-tailed student’s *t*-test, *t*(8) = 3.762, *P* = 0.0055].

**FIGURE 3:**
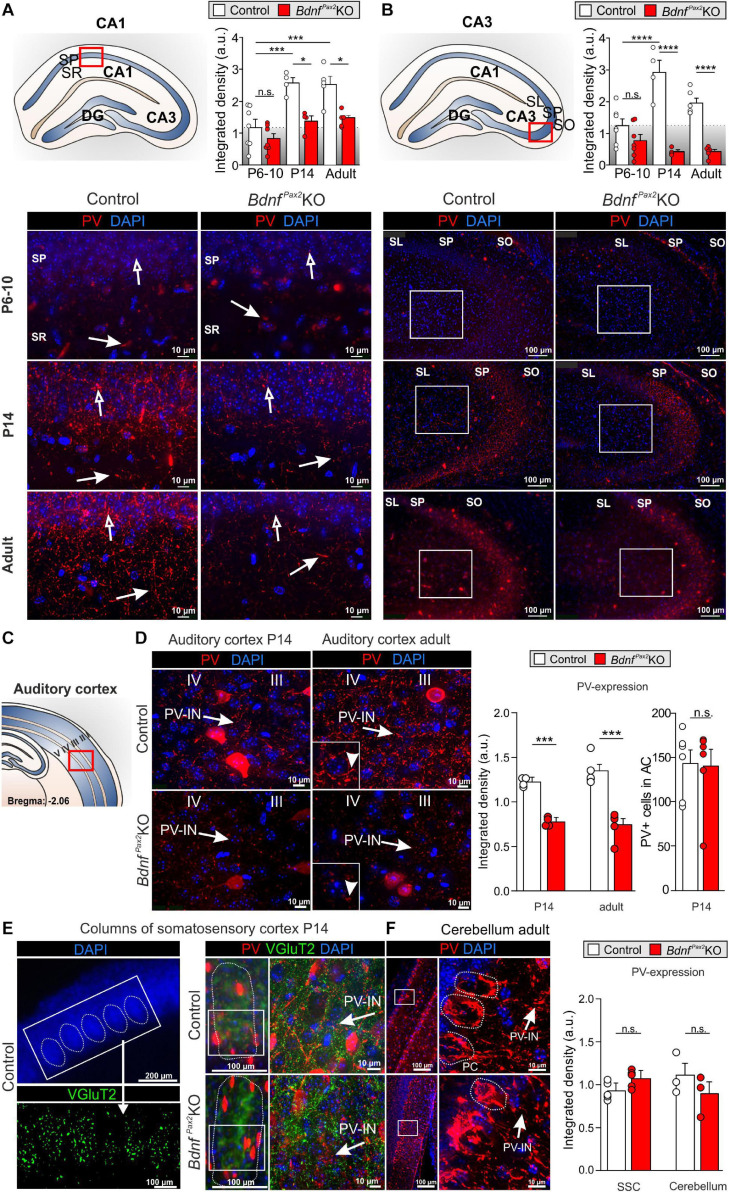
Parvalbumin immunostaining in *Bdnf^*Pax*2^*KOs during hearing onset. **(A)** Immunostaining of perisomatic PV (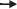) and dendritic PV (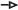) in the CA1 region during development. Scale bar = 10 μm. Right panel: Quantification of PV-IN fluorescence intensity in the CA1 region during development (*n* = 4-7 each, Genotype: *P* < 0.0001; Age: *P* < 0.0001). **(B)** Immunostaining of perisomatic PV (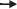) in the CA3 of adult *Bdnf^*Pax*2^*KOs and controls. Right panel: Quantification of the PV expression in the CA3 region (see white squares; *n* = 4-7 each, Genotype: *P* < 0.0001; Age: *P* < 0.05; Interaction: *P* < 0.001). **(C)** Scheme of the hippocampus, with CA1 and CA3 region and dentate gyrus (DG), and the AC with layers I to V. **(D)** Decreased expression of perisomatic (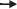) and dendritic (▶) PV-IN in the AC of adult *Bdnf^*Pax*2^*KOs (*n* = 5 each; *P* < 0.001) and P14 *Bdnf^*Pax*2^*KOs (*n* = 4 each; *P* < 0.001), in comparison to controls, while no difference in number of PV + cells in the adult AC was seen (*n* = 6 each; *P* > 0.05). **(E)** Left panel, exemplary picture for SSC columns. Middle panel: Normal development of columns in the SSC of P14 *Bdnf^*Pax*2^*KOs (red: PV; green: vGluT2). Left panel: Similar PV expression in SSC of P14 *Bdnf^*Pax*2^*KOs (*n* = 5 each; *P* = 0.303), compared to controls. **(F)** Normal PV expression was observed in the Cb of *Bdnf^*Pax*2^*KOs (*n* = 3 each; *P* = 0.331). Nuclear staining DAPI (blue). Scale bar **(A,B,D–F)** right panels: 10 μm; **(E)** lower left and middle panels, **(F)** left panel: 100 μm; **(E)** upper panel: 200 μm. Mean ± S.E.M. n.s. = *P* > 0.05, * = *P* < 0.05, *** = *P* < 0.001, **** = *P* < 0.0001.

Therefore, although normal numbers of PV-INs were observed in *Bdnf^*Pax*2^*KOs they showed reduced staining in their dendrites parallel to increased Arc levels. To explore to what extent this imbalance in inhibitory/excitatory markers may be the result of disturbed sculpting of PV-IN neurons by sensory experience, we analyzed the labeling of PV-INs prior to the critical plasticity period – between P6-P10, when in rodents sensory functions are still immature (de Villers-Sidani et al., 2007) – and toward its end (P14), as well as in adults (Figure 3).

In controls, between P6-P10 and at P14, we observed a significant elevation of PV protein staining in hippocampal regions, as shown for CA1 [Figure 3A; 2-way ANOVA, Genotype: *F*(1,26) = 27.10, *P* < 0.0001, Age: *F*(2,26) = 17.45, *P* < 0.0001, *n* = 4-7 mice] and CA3 regions [Figure 3B; 2-way ANOVA, Genotype: *F*(1,28) = 80.16, *P* < 0.0001, Age: *F*(2,28) = 5.049, *P* < 0.05, Interaction: *F*(2,28) = 11.76, *P* = 0.0002, *n* = 4-7 mice]. In *Bdnf^*Pax*2^*KOs, at P6-P10, the level of PV protein staining was not different from controls (Figures 3A,B; Bonferroni’s *post hoc* test: CA1: *P* > 0.05, CA3: *P* > 0.05, *n* = 7 mice each). However, at P14 and in adults, the levels of PV protein in *Bdnf^*Pax*2^*KOs remained low [Figures 3A,B; lower panels; Bonferroni’s *post hoc* test: P14: CA1: *P* < 0.05, CA3: *P* < 0.0001, *n* = 4 mice each, adult: CA1: *P* < 0.05, CA3: *P* < 0.0001, *n* = 4 (control) or 5 (*Bdnf^*Pax*2^*KO) each]. These observations indicate a significantly attenuated maturation of dendritic outgrowth of PV-INs in *Bdnf^*Pax*2^*KOs. This was also shown for the AC at P14 [Figures 3C,D left panel; unpaired two-tailed student’s *t*-test, *t*(6) = 6.229, *P* = 0.0008, *n* = 4 mice each] and in adults [Figures 3C,D, right panel; unpaired two-tailed student’s *t*-test, *t*(8) = 6.165, *P* = 0.0003, *n* = 4 mice each].

In order to explore if lower PV-IN labeling in the AC and hippocampus of *Bdnf^*Pax*2^*KOs may be associated with specific sensory modalities, PV labeling was also analyzed in the somatosensory cortex (Figure 3E) and Cb (Figure 3F). At P14, a time point for specific refinement of sensory coding in the barrel cortex (van der Bourg et al., 2017), sections were co-labeled for PV (Figure 3E, red) and the vesicular glutamate receptor 2 **(vGluT2**; Figure 3E, green), used to follow proper column formation (Sun, 2009). No difference between PV-IN levels was observed between controls and *Bdnf^*Pax*2^*KOs (Figure 3E). Also for the Cb, staining for PV-INs was not significantly different between controls and *Bdnf^*Pax*2^*KO mice [Figure 3F; unpaired two-tailed student’s *t*-test, *t*(4) = 1.104, *P* = 0.3314, *n* = 3 mice each]. This result suggests that the reduced PV-IN labeling intensity in *Bdnf^*Pax*2^*KOs may not be a common feature of all sensory cortices.

**In conclusion**, deletion of BDNF in Pax2-lineage descendants in brainstem regions results in diminished PV-IN labeling independently from the number of PV-INs in the AC and in the hippocampus from the end of the critical period at P14 onward. From P14 onward, reduced dendritic PV-IN labeling and elevated Arc levels are observed.

### *Bdnf^*Pax*2^*KOs Exhibit Elevated LTP and Reduced LTD

**FIGURE 4:**
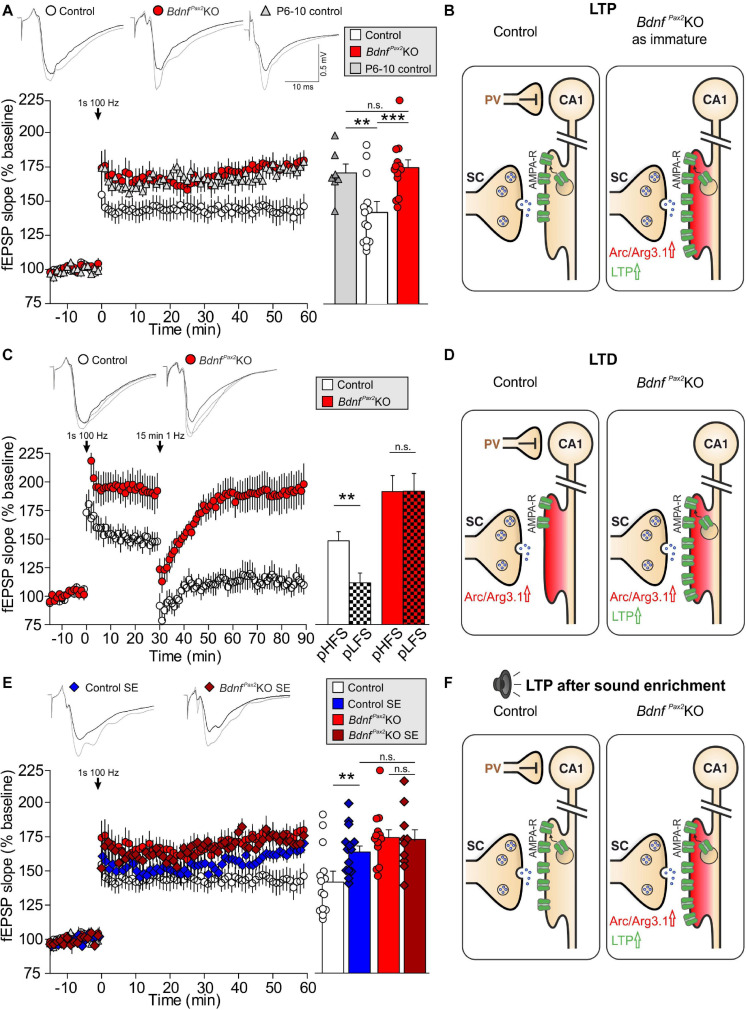
Hippocampal LTP/LTD adjustment in *Bdnf^*Pax*2^*KOs. **(A**,**C,E)** Upper panels, Averaged time courses of fEPSP slopes in acute brain slices from controls, *Bdnf^*Pax*2^*KOs, P6-10 controls, sound-exposed (SE) controls, and *Bdnf^*Pax*2^*KOs. Representative traces before (black) and after (gray) induction of LTP and after LTD induction (dotted black line) are shown. **(A)** Right panel: Higher LTP in adult *Bdnf^*Pax*2^*KOs (*n* = 9/13 animals/slices; 175.5 ± 5.7%) and P6-10 controls (*n* = 6/7 animals/slices; 171.6 ± 6.5%) compared with adult controls (*n* = 9/14 animals/slices; 142.4 ± 8.2%; *P* < 0.0001). **(B)** *Bdnf^*Pax*2^*KOs exhibit increased LTP due to reduced tonic PV inhibition. SC = Schaffer’s collateral. **(C)** Right panel: Successful LTP (*n* = 6/8 animals/slices; 148.9 ± 8.0%) and LTD (*n* = 6/8 animals/slices; 111.8 ± 8.6%) was observed for controls. *Bdnf^*Pax*2^*KOs had a significantly increased LTP (*n* = 6/10 animals/slices; 192.2 ± 14.1%) but did not show LTD maintenance (*n* = 6/10 animals/slices; 192.4 ± 15.7%) (control: *P* < 0.0001, *Bdnf^*Pax*2^*KO: *P* < 0.001). **(D)** *Bdnf^*Pax*2^*KOs exhibit impaired homeostatic plasticity to reduce synaptic strength. **(E)** Right panel: increased LTP was observed after SE (80-100 dB) controls (*n* = 4/11 animals/slices; 164.9 ± 5.9%) compared to unexposed controls (*n* = 9/14 animals/slices; 142.4 ± 8.2%) but not in SE *Bdnf^*Pax*2^*KOs (*n* = 3/5 animals/slices; 172.9 ± 12.9%) compared to unexposed *Bdnf^*Pax*2^*KOs (*n* = 9/14 animals/slices; 175.5 ± 5.7%; *P* < 0.0001). **(F)** *Bdnf^*Pax*2^*KOs exhibit impaired synaptic scaling properties and are not able to increase LTP after SE. Mean ± S.E.M. n.s. = *P* > 0.05, ** = *P* < 0.01, *** = *P* < 0.001.

Reduced labeling of dendrites in PV-INs linked to elevated Arc levels may indicate impaired integration of inhibitory PV-INs of the AC into functional hippocampal circuits. To test proper hippocampal function of *Bdnf^*Pax*2^*KOs, we first analyzed LTP by recording field excitatory postsynaptic potentials **(fEPSP**s) from acute forebrain slices at the CA3 to CA1 Schaffer’s collateral in the stratum radiatum. Interestingly, compared to controls, in *Bdnf^*Pax*2^*KOs the elevated Arc levels were linked to significantly higher fEPSP amplitudes, observed after high-frequency stimulation (1 s, 100 Hz) of the Schaffer’s collateral over the entire recording time period of 60 min (Figure 4A, left panel). Calculation of the mean of the last 10 min showed significantly higher LTP in *Bdnf^*Pax*2^*KOs [Figure 4A, right panel, white vs. red bar; 1-way ANOVA, *F*(5,62) = 42.81, *P* < 0.0001, control: *n* = 9/14, *Bdnf^*Pax*2^*KO: *n* = 9/13, P6-P10 controls: *n* = 6/7 mice/slices, Bonferroni’s *post hoc* test: *P* < 0.001]. To examine whether elevated fEPSP amplitudes in *Bdnf^*Pax*2^*KOs may be linked to the prevailing immaturity of the hippocampal PV-IN network (Figure 3), fEPSP slopes were also determined in acute brain slices from P6-P10 mice prior to the critical period when major sensory functions develop in rodents (de Villers-Sidani et al., 2007). We found that LTP levels were significantly higher in young (P6-P10) animals relative to adult controls (Figure 4A, left panel, gray bar; Bonferroni’s *post hoc* test: *P* < 0.01). In fact, the LTP levels of P6-P10 animals were comparable to the levels observed in adult *Bdnf^*Pax*2^*KO mice (Bonferroni’s *post hoc* test: *P* > 0.05), suggesting that immaturity of hippocampal PV-INs is reflected by elevated LTP.

It is conceivable that the elevated Arc levels in *Bdnf^*Pax*2^*KO mice may be due to insufficient shaping of synaptic contacts between PV-INs and pyramidal neurons through persistent (tonic) inhibition (Figure 4B). Since stimulus-induced endocytosis of postsynaptic AMPA receptors is required for the weakening of synapses during LTD (Jakkamsetti et al., 2013; Okuno et al., 2018), we next analyzed LTD responses in *Bdnf^*Pax*2^*KOs. In controls, high-frequency stimulation (1s, 100 Hz) led to elevated fEPSPs (LTP) [Figure 4C, white clear bar; 1-way ANOVA, *F*(2,21) = 13.59, *P* < 0.0001, *n* = 6/8 mice/slices, Bonferroni’s *post hoc* test: baseline/tetanized: *P* < 0.001] and subsequent low-frequency stimulation (15 min, 1  Hz) caused a reduction of fEPSP levels (LTD) back to baseline (Figure 4C, white patterned bar; Bonferroni’s *post hoc* test: tetanized/LFS: *P* < 0.01). In contrast, in *Bdnf^*Pax*2^*KOs, high-frequency stimulation led to even higher fEPSPs [Figure 4C, red clear bar; 1-way ANOVA, *F*(2,27) = 18.44, *P* < 0.001, *n* = 6/10 mice/slices, Bonferroni’s *post hoc* test: baseline/tetanized: *P* < 0.001] but a subsequent low-frequency stimulation (15 min, 1Hz) did not cause a reduction to baseline levels (Figure 4C, red patterned bar; Bonferroni’s *post hoc* test: tetanized/LFS: *P* > 0.05). These results suggest that the high levels of Arc in *Bdnf^*Pax*2^*KOs may have prevented a weakening of synapses and thus counteracted the generation of LTD (Figure 4D).

To get a first insight into whether these LTP/LTD changes in hippocampal slices have an impact on sensory function, we tested whether enriching sensory stimulation would enhance LTP in *Bdnf^*Pax*2^*KOs, as previously shown to occur in control mice 14 days after 40 min of SE at 80 dB SPL (Matt et al., 2018). Analysis of any LTP adjustment following SE at 80 dB SPL revealed significantly elevated LTP in the CA1 region of control animals [Figure 4E, blue bar; 1-way ANOVA; *F*(7,78) = 37.24, *P* < 0.0001, control: *n* = 9/14, control SE: *n* = 4/11, *Bdnf^*Pax*2^*KO: *n* = 9/14, *Bdnf^*Pax*2^*KO SE: *n* = 3/5 mice/slices; Bonferroni’s *post hoc* test: control/control SE: *P* < 0.05]. In contrast, in *Bdnf^*Pax*2^*KOs, the initial LTP responses were maintained (Figure 4E, red bar) and were not further elevated following SE (Figure 4E, dark red bar; Bonferroni’s *post hoc* test: control/control SE: *P* > 0.05). This finding is best explained by the inability of *Bdnf^*Pax*2^*KOs to adjust Arc levels in the postsynaptic spines of hippocampal pyramidal CA1 neurons, caused by impaired AMPA receptor trafficking (Diering and Huganir, 2018; Park, 2018; Figure 4F). Importantly, in response to a range of input strengths, *Bdnf^*Pax*2^*KOs and controls displayed similar levels of fEPSP amplitudes [Supplementary Figure 1A; 1-way ANOVA, *F*(4,25) = 0.80, *P* = 0.54, control: *n* = 9/14, control SE: *n* = 4/11 mice/slices, *Bdnf^*Pax*2^*KO SE: *n* = 3/5, *Bdnf^*Pax*2^*KO: *n* = 9/13, P6-P10 control: *n* = 6/7 mice/slices], and paired-pulse facilitation [Supplementary Figure 1B; 1-way ANOVA, *F*(4,25) = 0.49, *P* = 0.75, control: *n* = 9/14, control SE: *n* = 4/11 mice/slice, *Bdnf^*Pax*2^*KO SE: *n* = 3/5, *Bdnf^*Pax*2^*KO: *n* = 9/13, P6-P10 control: *n* = 6/7 mice/slices]. This indicates that the observed differences are not due to changes in presynaptic function, and suggests a normal activity of pre-synaptic Schaffer’s collaterals in *Bdnf^*Pax*2^*KOs.

**In conclusion**, the levels of hippocampal fEPSPs were elevated in *Bdnf^*Pax*2^*KOs, resembling the levels that were observed in controls prior to the critical developmental period of sensory onset between P10-P14. In adult *Bdnf^*Pax*2^*KOs the increased hippocampal fEPSPs is linked with elevated Arc and LTP levels, diminished LTD and deficits in LTP/LTD adjustment to enriching SE.

### *Bdnf^*Pax*2^*KOs Exhibit Diminished Learning, Reduced Exploratory Activity, and Enhanced Anxiety

**FIGURE 5:**
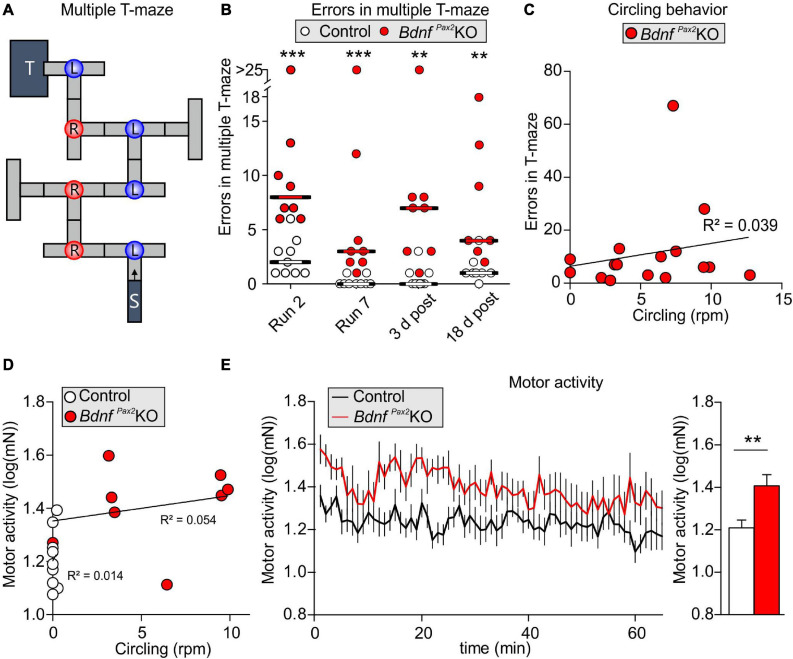
Learning, circling behavior and motor activity in *Bdnf^*Pax*2^*KOs. **(A)** The learning experiment was conducted in a multiple T-maze with 7 decision points (L, left turn correct; R, right turn correct). **(B)** *Bdnf^*Pax*2^*KOs had a significantly higher median error rate in all evaluated runs (*n* = 8/9 mice; *P* = 0.002), but showed successful learning from run 2 to run 7. However, long-term memory 3 and 18 days after the last training run was impaired in *Bdnf^*Pax*2^*KOs compared with controls. **(C)** Most *Bdnf^*Pax*2^*KOs displayed circling behavior; this circling behavior was not significantly correlated with learning errors (*Bdnf^*Pax*2^*KOs: *n* = 8; data shown for run 2 and 7). **(D)** While *Bdnf^*Pax*2^*KOs displayed more circling as well as more motor activity than controls, there was no significant correlation between these two measures within the two genotypic groups (*n* = 8/9 each). **(E)** Motor activity, measured on a ballistic platform during a startle measurement (*n* = 7-9 each; *P* = 0.007), was increased in *Bdnf^*Pax*2^*KOs. ** = *P* < 0.01, *** = *P* < 0.001.

Reduced dendritic labeling of PV-INs linked to elevated Arc levels and impaired LTP/LTD adjustment in *Bdnf^*Pax*2^*KOs may influence learning and behavioral processes. To approach this issue, a learning paradigm was used in which adult mice were trained to complete a maze in order to get a reward (access to their own mouse house). During this process, the mice had to find their way through the maze, memorizing 7 decision points in a multiple T-maze (Figure 5A). After completion of a successful run, the learning performance was analyzed by determining errors at the decision points of the maze. As shown in Figure 5B, in the four runs analyzed, the *Bdnf^*Pax*2^*KOs had a significantly higher error rate, making 1-67 errors at the end of the learning phase (run 7), while the controls made only 0-1 errors [Wilcoxon/Kruskal-Wallis-Tests, *X*^2^(1, *n* = 8/9) = 12.2753, *P* = 0.0005, control: *n* = 9 mice, *Bdnf^*Pax*2^*KO: *n* = 8 mice]. As most *Bdnf^*Pax*2^*KOs displayed circling behavior (Zuccotti et al., 2012), the correlation between circling behavior and motor activity or errors in the T-maze was explicitly tested. The circling behavior had neither an effect on the number of errors during runs 2 and 7 in the T-maze (Figure 5C; linear regression; *R*^2^ = 0.039, *n* = 8 mice), nor on the motor activity (Figure 5D; linear regression; control: *R*^2^ = 0.014, *Bdnf^*Pax*2^*KO: *R*^2^ = 0.054, control: *n* = 7 mice, *Bdnf^*Pax*2^*KO: *n* = 9 mice) that was significantly increased in *Bdnf^*Pax*2^*KOs, as measured on a ballistic platform in the startle apparatus [Figure 5E; unpaired two-tailed student’s *t*-test, *t*(17) = 3.08, *P* = 0.007, control: *n* = 7 mice, *Bdnf^*Pax*2^*KO: *n* = 9 mice]. This indicates that the increased learning errors in *Bdnf^*Pax*2^*KOs can be linked to neither circling behavior nor altered motor activity.

**FIGURE 6:**
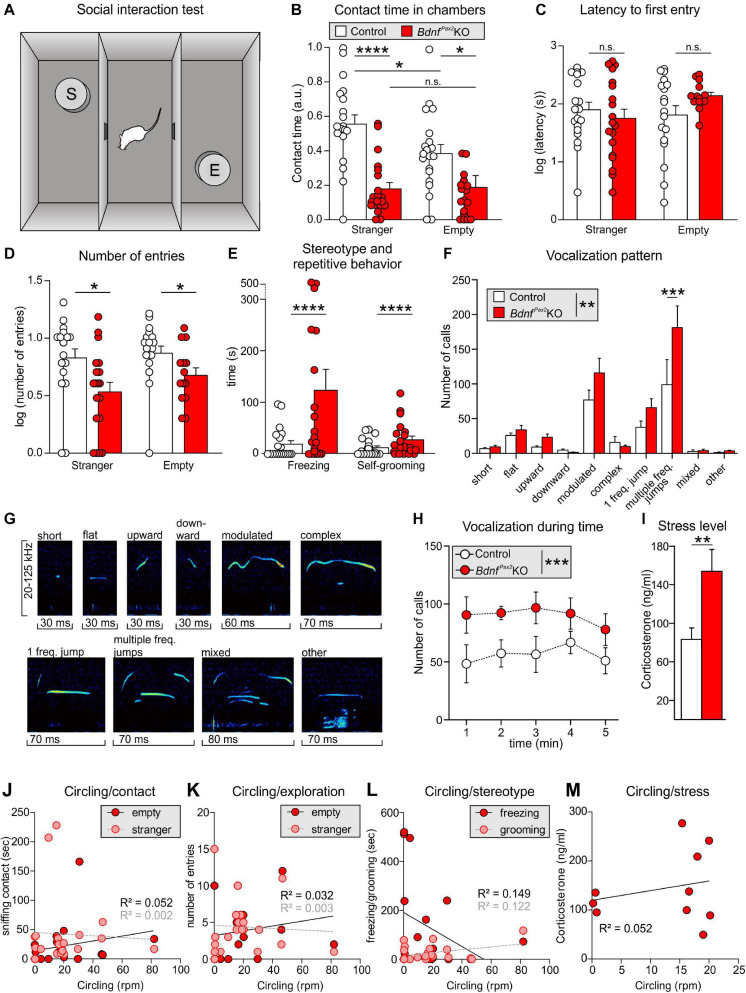
Social behavior and anxiety studies in *Bdnf^*Pax*2^*KOs. **(A)** Scheme of Crawley’s sociability apparatus with three chambers. In one of the outer chambers, a stranger mouse (S) was placed below the container while the other chamber was equipped with an empty (E) container. **(B)** *Bdnf^*Pax*2^*KOs spent less time with sniffing contacts toward both containers (*n* = 20 each, stranger: *P* < 0.0001; empty: *P* = 0.0278). Controls show a preference for the stranger which was not seen in *Bdnf^*Pax*2^*KOs (*n* = 20 each, control: *P* = 0.027; *Bdnf^*Pax*2^*KOs: *P* = 0.916). **(C)** The latency to the first entry of either outer compartment was similar for both genotypes [*n* = 14-19 each, stranger: *t*(36) = 0.70, *P* = 0.486; empty: *t*(30) = 1.68, *P* = 0.104]. **(D)** The number of entries into both chambers was decreased in *Bdnf^*Pax*2^*KOs (*n* = 14-19 each, stranger: *P* = 0.0138; empty: *P* = 0.0462). **(E)** *Bdnf^*Pax*2^*KOs revealed an increased stereotypic behavior shown by freezing (*n* = 20 each, *P* < 0.0001) and self-grooming (*n* = 20 each, *P* < 0.0001). **(F)** Differences in the vocalization pattern (shown in **G**) between *Bdnf^*Pax*2^*KO and control pups with increased multiple frequency jumps in *Bdnf^*Pax*2^*KOs (*n* = 8 each, Genotype: *P* = 0.004; Pattern: *P* < 0.0001; Interaction: *P* = 0.027). **(G)** Typical examples of vocalization patterns in mouse pups. **(H)** Ultrasonic call rate recorded in short-term isolated pups was increased in *Bdnf^*Pax*2^*KOs (*n* = 8 each; *P* < 0.001]. **(I)** Blood corticosterone level was increased in *Bdnf^*Pax*2^*KOs (*n* = 13 each, *P* = 0.048). **(J)** While *Bdnf^*Pax*2^*KOs displayed more circling as well as sniffing contact to any chamber than controls, there was no significant correlation between these two measures (*n* = 20). **(K)** Also no significant correlation between circling and exploration in both chambers, **(L)** their stereotypic behavior, namely freezing and grooming (*n* = 20), **(M)** or their endogenous stress level could be observed in *Bdnf^*Pax*2^*KOs (*n* = 8). **(C–H)** Mean ± S.E.M. n.s. = *P* > 0.05, * = *P* < 0.05, ** = *P* < 0.01, *** = *P* < 0.001, **** = *P* < 0.0001.

Altered Arc levels affecting AMPA receptor trafficking not only weaken preference and discrimination for novelty, but also affect anxiety and social behavior (Cheng et al., 2017; Penrod et al., 2019). To test for altered behavior, we used Crawley’s sociability 3-chamber test (Figure 6A) to analyze the social and explorative behaviors of controls and *Bdnf^*Pax*2^*KOs. The time of sniffing contacts toward an empty cage or a cage with an unknown (“stranger”) mouse was monitored and normalized to the time spent in the respective chamber. Controls spent more time sniffing toward the stranger-mouse chamber than toward the empty chamber, while *Bdnf^*Pax*2^*KOs showed no preference between the two [Figure 6B; control: unpaired two-tailed student’s *t*-test, *t*(38) = 2.29, *P* = 0.027, *Bdnf^*Pax*2^*KO: unpaired two-tailed student’s *t*-test, *t*(18) = 0.11, *P* = 0.916, *n* = 20 mice each]. Furthermore, *Bdnf^*Pax*2^*KOs differed from controls in showing significantly reduced sniffing contacts toward both cages [Figure 6D; empty: unpaired two-tailed student’s *t*-test, *t*(38) = 5.84, *P* = 0.0278, stranger: unpaired two-tailed student’s *t*-test, *t*(38) = 5.84, *P* < 0.0001, *n* = 20 mice each], although the average latency for the first entry into the empty chamber or chamber with a stranger was not different between controls and *Bdnf^*Pax*2^*KOs [Figure 6C; empty: unpaired two-tailed student’s *t*-test, *t*(30) = 1.68, *P* = 0.0205, stranger: unpaired two-tailed student’s *t*-test, *t*(36) = 0.70, *P* = 0.486, *n* = 20 mice each]. Moreover, in comparison to controls, *Bdnf^*Pax*2^*KOs exhibited significantly fewer entries into both chambers [Figure 6F; empty: unpaired two-tailed student’s *t*-test, *t*(30) = 2.08, *P* = 0.0462, stranger: unpaired two-tailed student’s *t*-test, *t*(36) = 2.59, *P* = 0.0138, *n* = 20 mice each]. This suggests that *Bdnf^*Pax*2^*KOs either show an altered behavioral reactivity during a novel situation, or develop diminished consolidation of newly learned information, both of which crucially influence stress and anxiety responses (de Kloet et al., 1999). Anxiety can be assessed through altered grooming or corticosterone levels (Kromer et al., 2005). When analyzing freezing or self-grooming behaviors, *Bdnf^*Pax*2^*KOs showed a significant increase in spontaneous freezing [Figure 6G, left side; Chi-square test for trend, *X*^2^(1, *n* = 20 each) = 199.8, *P* < 0.0001] and self-grooming behaviors [Figure 6G, right side; Chi-square test for trend, *X*^2^(1, *n* = 20 mice each) = 24.5, *P* < 0.0001].

The next measure was of the ultrasound vocalization (**USV**) of nursing infants at P7. This revealed significant differences in the vocalization patterns between control and *Bdnf^*Pax*2^*KO pups, as depicted in Figures 6F,G. USV with multiple frequency jumps were more frequent in *Bdnf^*Pax*2^*KO pups (Figure 6F, *n* = 8 mice each, Genotype: *P* = 0.004). Additionally, isolated *Bdnf^*Pax*2^*KO pups showed increased numbers of all USV calls during a 5 min period (Figure 6H, *n* = 8 mice each; *P* < 0.0001), which indicated a higher index of anxiety (Kromer et al., 2005; Groenink et al., 2008). In adult *Bdnf^*Pax*2^*KOs, basal corticosterone levels were significantly elevated compared to controls [Figure 6I; unpaired two-tailed student’s *t*-test, *t*(24) = 2.082, *P* = 0.0482, *n* = 13 mice each], also indicating increased anxiety behavior, a hallmark of stress.

With regard to *Bdnf^*Pax*2^*KOs displaying circling behavior (Chumak et al., 2016) and to suggest that specific vestibular dysfunction can cause learning and behavior deficits (Smith, 2019), we correlated circling with behavioral deficits in *Bdnf^*Pax*2^*KOs. Although *Bdnf^*Pax*2^*KOs displayed more circling and less sniffing contact to any chamber than controls, there was no significant correlation between these two measures in any chamber (Figure 6J; linear regression; empty: *R*^2^ = 0.052, stranger: *R*^2^ = 0.0022; *n* = 20 mice). There was also no significant correlation observed between circling and exploration in both chambers (Figure 6K; linear regression; empty: *R*^2^ = 0.0326, stranger: *R*^2^ = 0.0032; *n* = 20 mice), the circling and stereotypic behavior of freezing and grooming (Figure 6L; linear regression; freezing: *R*^2^ = 0.1491, grooming: *R*^2^ = 0.1218; *n* = 20 mice), or circling and the endogenous stress levels (Figure 6M; linear regression; *R*^2^ = 0.0521; *n* = 10 mice).

**Conclusion:** In *Bdnf^*Pax*2^*KOs reduced dendritic labeling of PV-INs and elevated Arc levels in the AC and hippocampus are associated with impaired LTP/LTD adjustment. How the attenuated capacity to memorize novel T-maze cues, the elevated anxiety, and the reduced social behavior, found in *Bdnf^*Pax*2^*KOs are causally linked with impaired LTP/LTD adjustment and imbalanced PV-IN and Arc levels remains to be determined.

### BDNF Deletion in Pax2-Lineage Descendants Led to Reduced Fine-Grained Auditory Brainstem Output Activity

**FIGURE 7:**
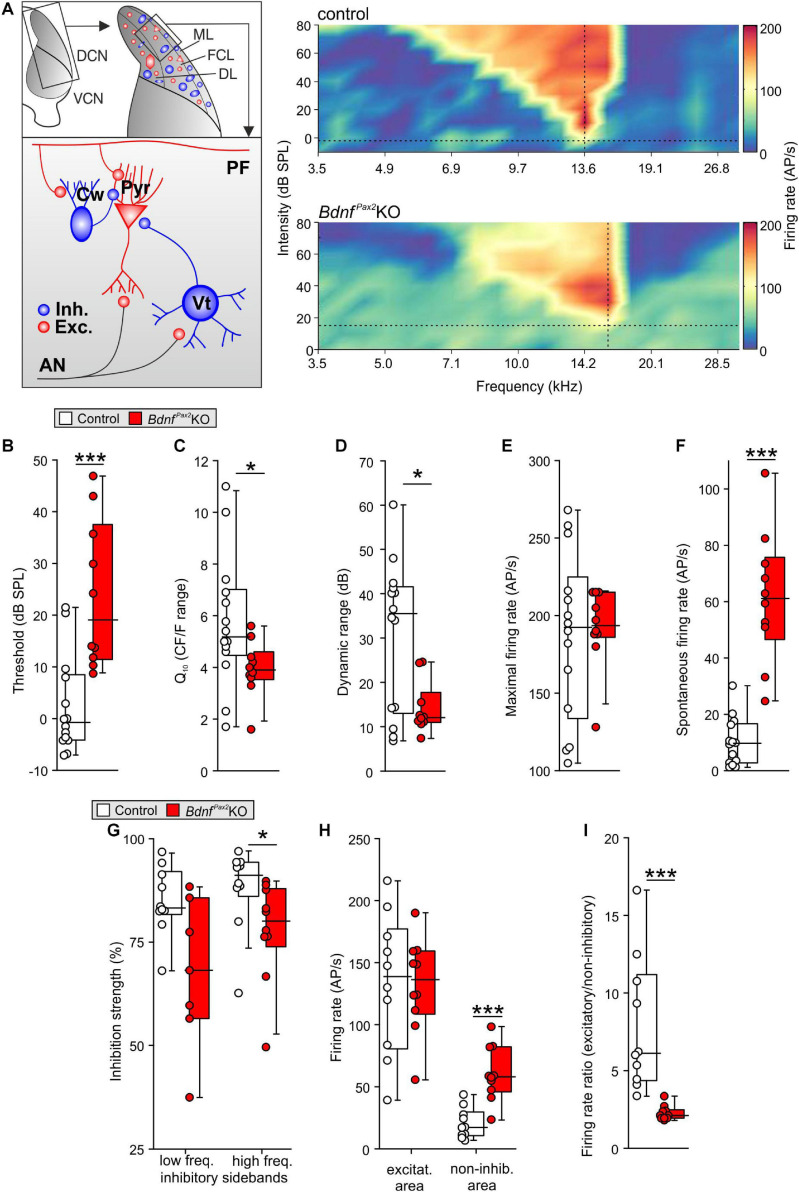
*In vivo* recordings in the dorsal cochlear nucleus of *Bdnf^*Pax*2^*KO. **(A)** Left: schematic drawing of neurons in the fusiform layer of the DCN. Right: Example for frequency response areas in DCN fusiform cells of controls (upper panel) and *Bdnf^*Pax*2^*KOs (lower panel) representing the neuronal AP discharge rate evoked by 100ms pure-tone stimulation with varying frequency-level combinations. Color scale indicates firing rate between 0 (dark blue) and 205 (dark red) APs per second. Dotted vertical lines = units’ characteristic frequency (CF), dotted horizontal lines = threshold levels. The frequency-level combinations evoking significant increase in firing rates form a characteristic V-shaped excitatory area (yellow to red). The regions with decreased activity form low- and high-frequency inhibitory sidebands (dark blue). Note the increased activity outside of the excitatory and inhibitory areas in *Bdnf^*Pax*2^*KOs. **(B)** Elevated thresholds of tone-evoked responses in *Bdnf^*Pax*2^*KOs in comparison to controls (*n* = 10-14; *P* < 0.001). **(C)** Quality factor (Q_10_), representing the relative bandwidth of the excitatory field 10 dB above threshold, revealed sharper frequency selectivity in controls compared to *Bdnf^*Pax*2^*KOs (*n* = 10-14; *P* = 0.048). **(D)** Dynamic ranges of rate-level function at units’ CF were significantly smaller in *Bdnf^*Pax*2^*KOs than in controls (*n* = 10-14; *P* = 0.038). **(E)** Maximal firing rates evoked by acoustic stimulation were comparable between the groups (*n* = 10-14; *P* = 0.86). **(F)** Spontaneous neuronal firing in *Bdnf^*Pax*2^*KOs was significantly increased compared to controls (*n* = 10-14; *P* < 0.001). **(G)** Inhibitory strength in low-frequency inhibitory sidebands (left) was similar, while high-frequency inhibitory sidebands (right) showed reduced inhibitory strength in *Bdnf^*Pax*2^*KOs (*n* = 10 each*;* low-frequency sidebands: *P* = 0.057, high-frequency sidebands: *P* = 0.021). **(H)** Action potential firing rates within units’ excitatory areas (left) were similar. Non-inhibitory areas, i.e., outside of excitatory area and inhibitory sidebands (right), displayed markedly increased firing rates in *Bdnf^*Pax*2^*KOs (*n* = 10 each; excitatory: *P* = 0.958, non-inhibitory: *P* < 0.001). **(I)** Ratios of AP firing rates between excitatory and non-inhibitory areas were smaller in *Bdnf^*Pax*2^*KOs than in controls (*n* = 10 each, *P* < 0.001). Box plots show medians, the 25 and 75 percentiles, and the interdecile ranges. Dots show values for individual cells. * = *P* < 0.05, *** = *P* < 0.001.

The reduced dendritic PV-IN labeling in the AC and hippocampus of *Bdnf^*Pax*2^*KOs from P10 onward (Figure 3) may suggest that BDNF in Pax2-lineage descendants in brainstem regions may be required to shape auditory brainstem activity in order to generate a proper auditory-specific driving force for thalamo-cortical integration of the GABAergic PV-IN network into functional fronto-striatal circuits (Wehr and Zador, 2003; Gabernet et al., 2005). Before testing for proper auditory brainstem responses (**ABR**), we confirmed near-normal hearing thresholds in *Bdnf^*Pax*2^*KOs (Zuccotti et al., 2012). Normal electromechanical response properties of OHCs and slightly diminished IHC exocytosis in high-frequency cochlear regions (Zuccotti et al., 2012) led only to a mild threshold elevation and reduced response amplitude of responses to clicks, noise bursts or pure tone stimuli [Supplementary Figure 2A; click: unpaired two-tailed student’s *t*-test, *t*(47) = 3.224, *P* = 0.0023, control: *n* = 26 mice, *Bdnf^*Pax*2^*KO: 23 mice, noise: unpaired two-tailed student’s *t*-test, *t*(47) = 2.306, *P* = 0.0256, control: *n* = 26 mice, *Bdnf^*Pax*2^*KO: 23 mice, f-ABR: 2-way ANOVA, Genotype: *F*(1,9) = 59.72, *P* < 0.0001, *n* = 16/32 mice/ears each]. Next, the sensitivity for auditory-specific stimuli was determined by analyzing brainstem neurons in the cochlear nucleus (**CN**) that are targeted by auditory nerve **(AN)** fibers (Figure 7A). Fusiform/pyramidal neurons in the DCN receive direct excitatory inputs onto their basal dendrites from the descending branch of the AN (Palombi et al., 1994; Zhou et al., 2015). AN inputs also activate inhibitory GABAergic vertical cells that project to the soma of the fusiform/pyramidal DCN neurons (Spirou et al., 1999; Figure 7A, left panel). Recordings from DCN neurons of adult *Bdnf^*Pax*2^*KO mice revealed elevated tone-evoked thresholds compared to control (Figure 7B; Mann-Whitney rank sum test, *U* = 11, *P* < 0.001, controls: *n* = 14 mice, *Bdnf^*Pax*2^*KO: 10 mice). Moreover, a broader tuning in frequency bandwidth, measured as a reduced quality factor (Q_10_) [Figure 7C; unpaired two-tailed student’s *t*-test, *t*(22) = 2.1, *P* = 0.048, control: *n* = 14 mice, *Bdnf^*Pax*2^*KO: 10 mice], and a decreased dynamic range (Figure 7D; Mann-Whitney rank sum test, *U* = 34, *P* = 0.038, control: *n* = 14 mice, *Bdnf^*Pax*2^*KO: 10 mice) were observed. While the maximal firing rate was not different between the two genotypes (Figure 7E; Mann-Whitney rank sum test, *U* = 66.5, *P* = 0.86, control: *n* = 14 mice, *Bdnf^*Pax*2^*KO: 10 mice), the spontaneous firing rate (**SFR**) was strongly increased in *Bdnf^*Pax*2^*KOs (Figure 7F; Mann-Whitney rank sum test, *U* = 1, *P* < 0.001, control: *n* = 14 mice, *Bdnf^*Pax*2^*KO: 10 mice). Furthermore, in *Bdnf^*Pax*2^*KOs, the inhibitory strength was reduced within high-frequency sidebands (Figure 7G right panel; Mann-Whitney rank sum test, *U* = 19, *P* = 0.021, control: *n* = 14 mice, *Bdnf^*Pax*2^*KO: 10 mice), but not within low frequency sidebands (Figure 7G left panel; Mann-Whitney rank sum test, *U* = 15, *P* = 0.057, control: *n* = 14 mice, *Bdnf^*Pax*2^*KO: 10 mice). Action-potential firing rates were similar for excitatory frequency response areas [Figure 7H left panel; unpaired two-tailed student’s *t*-test, *t*(18) = 0.053, *P* = 0.958, control: *n* = 14 mice, *Bdnf^*Pax*2^*KO: 10 mice]. In contrast, *Bdnf^*Pax*2^*KOs had strongly increased firing rates in non-inhibitory areas, i.e., outside of the excitatory area and the inhibitory sidebands [Figure 7H right panel; unpaired two-tailed student’s *t*-test, *t*(18) = 5.04, *P* < 0.001, control: *n* = 14 mice, *Bdnf^*Pax*2^*KO: 10 mice]. Comparison of the action-potential firing rates between excitatory and non-inhibitory areas of DCN neurons revealed a reduced ratio in *Bdnf^*Pax*2^*KOs (Figure 7I; Mann-Whitney rank sum test, *U* = 0, *P* < 0.001, control: *n* = 14 mice, *Bdnf^*Pax*2^*KO: 10 mice), indicating a tonically diminished inhibitory strength.

Elevated thresholds linked with elevated SFR, reduced inhibitory strength, and reduced dynamic range of sound-induced DCN responses identified a significantly diminished resolution of AN input. Altered summed AN activity, previously observed in *Bdnf^*Pax*2^*KOs (Chumak et al., 2016), was confirmed, demonstrating a reduced supra-threshold summation of ABR wave I [Supplementary Figure 2B; 2-way ANOVA; Genotype: *F*(1,18) = 199.0, *P* < 0.0001, control: *n* = 14/28 mice/ears, *Bdnf^*Pax*2^*KO: *n* = 12/24 mice/ears]. ABR wave I response patterns were found to be shortened in time [Supplementary Figure 2B, 2-way ANOVA; Genotype: *F*(1,17) = 187.50, *P* < 0.0001, control: *n* = 14/28 mice/ears, *Bdnf^*Pax*2^*KO: *n* = 12/24 mice/ears], possibly resulting from a degraded spike precision, a reduced reliability or a diminished synchronicity of AN responses. Whereas DCN first spike latencies were not altered [unpaired two-tailed student’s *t*-test, *t*(21) = 0.183, *P* = 0.857, control: *n* = 14 mice, *Bdnf^*Pax*2^*KO: 10 mice; data not shown], ABR wave IV was significantly delayed and reduced in amplitude in *Bdnf^*Pax*2^*KOs [Supplementary Figure 2C; amplitude: 2-way ANOVA; Genotype: *F*(1,18) = 15.76, *P* < 0.0001, latency: 2-way ANOVA; Genotype: *F*(1,17) = 78.96, *P* < 0.0001, control: *n* = 14/28 mice/ears, *Bdnf^*Pax*2^*KO: *n* = 12/24 mice/ears], confirming previous results (Chumak et al., 2016).

To explore possible temporal auditory processing deficits, we analyzed the coding of amplitude-modulated tones in auditory steady-state responses (**ASSR**s) by measuring the response to differently modulated stimuli. Compared to controls, we detected significantly reduced ASSRs at a modulation depth of more than 10% in *Bdnf^*Pax*2^*KOs [Supplementary Figure 2D, left panel; 2-way ANOVA; Genotype: *F*(1,15) = 10.92, *P* = 0.0011, *n* = 10 mice each], indicating severe deficits in temporal resolution. When these ASSRs were analyzed as a function of the stimulus level in a phase-locked manner, responses in *Bdnf^*Pax*2^*KO mice remained reduced, particularly for low sound pressure levels close to threshold [Supplementary Figure 2D, right panel; 2-way ANOVA; Genotype: *F*(1,14) = 28.15, *P* < 0.0001, *n* = 10 mice each], suggesting profound deficits in the fast temporal processing of sound signals near hearing threshold.

**FIGURE 8:**
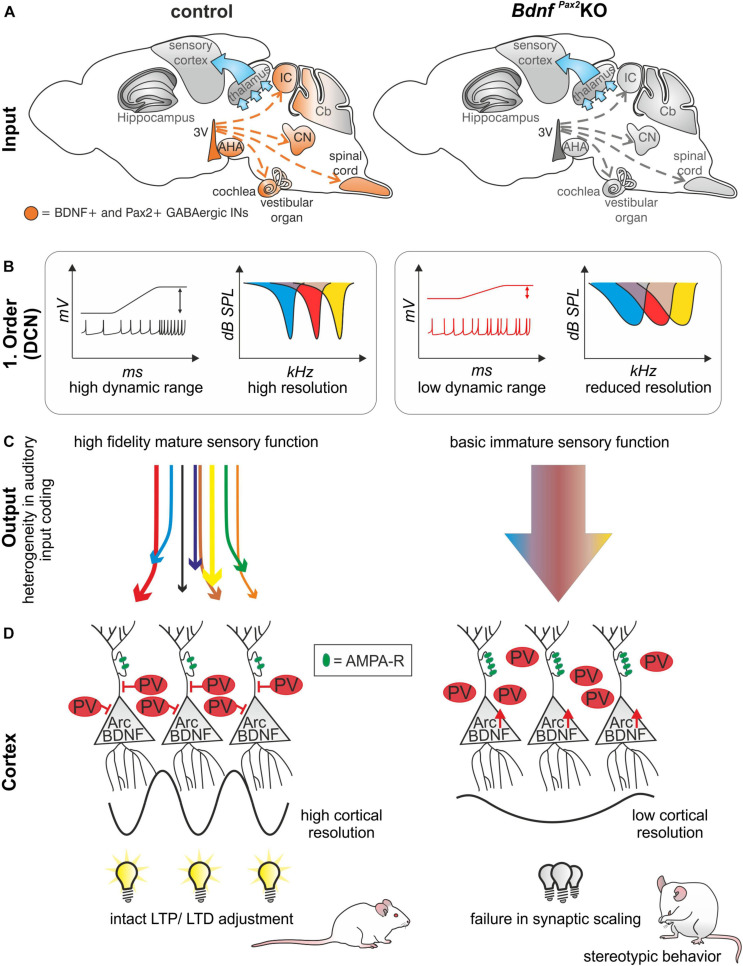
Schematic how BDNF in GABAergic IN precursors shapes sensory memory. **(A)** In *Bdnf^*Pax*2^*KOs, BDNF is deleted in Pax2-lineage descendants in the sensory periphery. **(B)** This leads to reduced dynamic range and reduced resolution in the first order neurons of *Bdnf^*Pax*2^*KOs. **(C)** Mature sensory systems develops only if BDNF is expressed in GABAergic IN precursors. **(D)** *Bdnf^*Pax*2^*KOs fail to mature dendritic outgrowth of PV-INs in the AC and hippocampus, resulting in impaired synaptic scaling in LTP and LTD and increased stereotypic behavior. AMPA-R = AMPA receptor.

**In conclusion:** The absence of BDNF in Pax2-lineage descendants in *Bdnf^*Pax*2^*KOs leads to elevated thresholds and reduced sound-evoked ABR amplitudes, linked with an increased SFR and a reduction of tonic inhibitory strength and dynamic range of sound-induced DCN responses (Figures 8A,B). Diminished fine-grained auditory input in *Bdnf^*Pax*2^*KO mice (Figure 8C) is associated with elevated baseline levels of Arc and reduced labeling of PV-INs, particularly in dendritic regions of the AC and hippocampus, while leaving the number of PV-INs unchanged (Figure 8D). The subsequent inability to adjust LTP/LTD may be causally linked with the attenuated capacity to memorize novel T-maze cues, elevated anxiety, and reduced social behavior, all of which are characteristic features of an autistic-like phenotype (Figure 8D).

## Discussion

The results of the present study suggest that the absence of BDNF in Pax2-lineage descendants in hindbrain regions in *Bdnf^*Pax*2^*KO mice may have an impact on learning and behavior through impaired activity-driven integration of the GABAergic PV-IN network of the AC into functional hippocampal circuits. As a result the observed deficits in stimulus-induced hippocampal LTP/LTD adjustments (social) learning, and anxiety control in *Bdnf^*Pax*2^*KOs may be caused by an inability to scale hippocampal synapses.

### BDNF Expression in Pax2-Lineage Descendants in Lower Auditory Hindbrain Regions

Here, BDNF mRNA was found to be present in Pax2-lineage descendants within brainstem regions, as well as the AHA area, but not in Pax2+ septo-hippocampal projection neurons, which modulate brain plasticity through AHA regions (Bakos et al., 2016), or in the Cb (Figure 1). Moreover, no overlap between tdTomato fluorescence and BDNF mRNA was found in any thalamic or cortical frontal brain region. The latter was expected, since GABAergic IN precursors, derived from Pax2-lineage descendants, are supposed to migrate mainly from ventricular zones to lower brain levels that are posterior to midbrain regions, including the Cb, spinal cord, and inner-ear regions (Nornes et al., 1990; Maricich and Herrup, 1999; Rowitch et al., 1999; Fotaki et al., 2008).

We did not observe BDNF expression in all Pax2-lineage descendants in brainstem/hindbrain regions between P10 and adults (Figure 1). However, BDNF may be transiently expressed in Pax2-lineage descendants before this stage. We thus cannot exclude that the early expression of BDNF in Pax2-lineage descendants in for instance the Cb may participate in inhibitory circuit formation at the level of basket, granule, and stellate cells (Collin et al., 2005) and thereby contributes to the elevated motor activity observed in *Bdnf^*Pax*2^*KOs. More detailed analysis should also focus on the spinal cord and vestibular nucleus, particularly regarding numerous studies that demonstrate dysfunctions of both may possibly have the potential to lead to spatial memory deficits and cognitive decline in humans (Smith, 2019). Finally, we cannot entirely exclude that, in addition to the deficits in fast auditory processing, subtle functional deficits may also exist in the somatosensory or visual system of *Bdnf^*Pax*2^*KOs, although we observed no apparent changes in inhibitory/excitatory balance within these systems. Here, more specific fine-structured testing would be required to further validate this aspect. In this context, tracing of BDNF+ cells in Pax2-lineage descendants may be required. While it is generally believed that BDNF mRNA transcripts are absent from inhibitory INs (Canals et al., 2001; Cohen-Cory et al., 2010; Andreska et al., 2014), the few studies that have observed BDNF in GABAergic IN precursors in hindbrain or cortical neurons (Jungbluth et al., 1997; Huang et al., 1999; Barreda Tomas et al., 2020) may be reconsidered in the light of the present data.

### Reduced Dendritic Outgrowth of PV-INs in Frontal Brain Regions in *Bdnf^*Pax*2^*KOs

Here, we demonstrated that PV-INs in *Bdnf^*Pax*2^*KOs were unaffected up to P10, at which point their numbers reached normal levels in cortical and hippocampal regions (Figure 2). This indicates that subpallium-derived GABAergic neurons have most likely reached their cortical and hippocampal target regions in *Bdnf^*Pax*2^*KOs, a process shown to be accomplished by the 2nd postnatal week in rodents (Marin and Rubenstein, 2001). Between P10 and P14, however, dendritic growth of PV-INs in the AC and hippocampus remained significantly diminished in *Bdnf^*Pax*2^*KO mice, although BDNF mRNA expression was maintained at levels comparable to those of control mice (Figure 3).

During the critical period of sensory system maturation, neuronal activity (due to sensory experience) is likely to be important, not only for the termination of the migration of cortical INs, but also for their proper integration into functional circuits (de Villers-Sidani et al., 2007; Lim et al., 2018). There is agreement that an activity-dependent release of BDNF from pyramidal neurons is required to sculpt the integration of the cortical PV-IN network in nearly all sensory cortices, probably by driving cortical tonic inhibition through synaptogenesis of peri-somatic PV-INs with pyramidal neurons (Hong et al., 2008; Xu et al., 2010; Griffen and Maffei, 2014; Lim et al., 2018; Meis et al., 2019). The proper integration of GABAergic INs into higher cortical sensory regions is essential for proper feed-forward inhibition, the sharpening of receptive fields, and pattern separation (Pouille and Scanziani, 2001; Leutgeb et al., 2007). Only recently, it was shown that the process of GABA-IN dendritic synaptogenesis with pyramidal neurons during network integration may be linked with an upregulation of the potassium chloride cotransporter 2 (**KCC2**) in INs, which halts the motility of INs by gradual reduction of the frequency of spontaneous intracellular calcium transients in response to GABA (Bortone and Polleux, 2009), causing an excitatory-to-inhibitory switch in GABAergic signaling (Marin and Rubenstein, 2001; Ben-Ari, 2002). In the auditory system, the GABAergic excitatory-to-inhibitory switch occurs in a region-specific pattern after hearing onset (Kandler and Friauf, 1995; Friauf et al., 2011), possibly driven by sensory experience (Shibata et al., 2004). Previous findings suggest a crucial temporally and spatially heterogeneous role of KCC2 and BDNF for filopodia extensions of GABAergic INs during cortical maturation (Awad et al., 2018). When for example KCC2 is removed in immature cortical neurons spine maturation was prevented altogether, leading to an increase of filopodia protrusions (Li et al., 2007). BDNF is one of the strongest modulators of KCC2 activity (Wardle and Poo, 2003; De Koninck, 2007; Kaila et al., 2014). Likewise, BDNF is an activity-driven gene (West et al., 2001, 2014) that was previously suggested to require fast auditory processing in order to be recruited for memory-dependent adjustments of LTP following SE (Matt et al., 2018). Nearly normal basal hearing thresholds of *Bdnf^*Pax*2^*KOs (Zuccotti et al., 2012; Chumak et al., 2016) which are also observed in the present study (Figure 7), along with reduced and delayed ABR wave IV responses indicate that basic sound processing through auditory fibers with a low spontaneous firing rate (**SR**) and high activation thresholds (Merchan-Perez and Liberman, 1996) that develop early at hearing onset (Glowatzki and Fuchs, 2002; Grant et al., 2010) is intact in *Bdnf^*Pax*2^*KOs. On the other hand, our results strongly suggest that high-SR auditory fibers with low activation thresholds that develop only after hearing onset (Glowatzki and Fuchs, 2002; Grant et al., 2010) are underdeveloped in *Bdnf^*Pax*2^*KOs. These fibers define the detection thresholds for sounds and the shortest latencies at any given characteristic frequency (Meddis, 2006; Heil et al., 2008; Bourien et al., 2014). Diminished fast (high-SR) auditory processing would best explain not only the reduced and delayed ABR wave IV responses, but also the reduced activation of fusiform/pyramidal DCN neurons in *Bdnf^*Pax*2^*KOs. DCN neurons are directly activated through AN fibers (Palombi et al., 1994; Zhou et al., 2015). The diminished high-SR AN activity may thus explain elevated thresholds, broadened bandwidth, reduced high-frequency sideband inhibition, and elevated spontaneous firing rates of DCN neurons in *Bdnf^*Pax*2^*KOs. Accordingly, less inhibitory shaping of AN responses or attenuated shaping of inhibitory GABAergic vertical cells that contact the soma of DCN neurons (Spirou et al., 1999) may explain the phenotype of DCN neurons in *Bdnf^*Pax*2^*KOs. Regarding the previously observed diminished filopodia extensions of GABAergic INs observed upon KCC2 deletion (Awad et al., 2018), we may consider diminished filopodia extension of GABAergic IN during development to occur when driving force for KCC2 upregulation is too low. Therefore, if fast auditory-specific processing is too low or unspecific to promote activity-dependent BDNF-driven KCC2 upregulation in ascending auditory and associated fronto-striatal networks, it is likely that PV-IN filopodia extensions of GABAergic INs would not mature properly, as observed here in the AC and hippocampus of *Bdnf^*Pax*2^*KO mice (Figures 2, 3).

The crucial requirement for the proper integration of GABAergic INs into functional circuits is best documented by studies that demonstrated a dysfunction in subpallium-derived GABAergic migration processes that are suggested to lead to neurodevelopmental disorders, including ASD (Marin, 2012; Canetta et al., 2016; Skene et al., 2018).

The present study indicates that not only the dysfunction of subpallium-derived GABAergic migration processes, but also defects in GABAergic IN precursors migrating to lower hindbrain regions, can lead to neurodevelopmental disorders, including ASD. The latter process may provide the proper driving force for the former one, a dependency the brain cannot compensate for when deficient.

### *Bdnf^*Pax*2^*KOs Exhibit Diminished PV-IN Dendritic Outgrowth Linked With Impaired Executive Functions

Reduced PV-IN dendrites in *Bdnf^*Pax*2^*KOs in cortical and hippocampal regions, coinciding with elevated levels of Arc mRNA and protein (Figures 2, 3), suggest that the synaptic activity of PV-IN contacts with pyramidal neurons may lower the baseline of Arc levels in pyramidal neurons. The expression of Arc varies with brain regions. While Arc is present in excitatory neurons in the hippocampus and the primary cortex, in for example the dorsal striatum Arc can also be found in projection GABAergic medial septal neurons (Vazdarjanova et al., 2006; Gong et al., 2020). The preferential expression in glutamatergic excitatory neurons in the hippocampus and the change in number of Arc+ neurons and expression level with strength of stimuli in the hippocampus (Link et al., 1995; Guzowski et al., 2006; Vazdarjanova et al., 2006; Bramham et al., 2008; Zhang and Bramham, 2020) are consistent with the observations that **(i)** Upon glutamate-induced stimulation of projection neurons, expression of Arc is initiated remarkably quickly (∼15 s), leading to an elevation of its levels, which in turn induces a rapid removal of postsynaptic AMPA receptors and thereby weakens the synapse (Waung et al., 2008). If Arc baseline levels persist in a saturated stage because the resting potential of pyramidal neurons is not shaped through PV-IN inhibition, as hypothesized here for *Bdnf^*Pax*2^*KOs (Figures 2D,F), postsynaptic spines of CA1 pyramidal neurons respond to high-frequency stimulation with elevated fEPSP levels as observed in the present study and also prior to hearing onset (Figure 6A). **(ii)** Moreover, consecutive stimulations at low-frequencies in *Bdnf^*Pax*2^*KOs did not bring fEPSP levels back to baseline through LTD (Figure 6C), suggesting its incapacity to further elevate Arc levels and weaken synapses (Waung et al., 2008). **(iii)** SE at 80dB SPL typically leads to continuously elevated LTP in control animals (Matt et al., 2018), but not in *Bdnf^*Pax*2^*KOs (Figure 6E), suggesting that the positive-feedback cycle predicted to be required to amplify specific sensory stimuli during improved task-performance (Irvine, 2018) does not work properly. **(iv)** Based on the concept that novelty discrimination crucially depends on proper AMPA receptor trafficking in postsynaptic spines, which leads to the rapid weakening of synapses and LTD formation (Waung et al., 2008), the cognitive deficits of *Bdnf^*Pax*2^*KOs in the multiple T-maze (Figures 5A,B) may be a consequence of inappropriate AMPA receptor trafficking in the postsynaptic spines of pyramidal neurons. This is due to saturated Arc baseline levels that, before lower baseline levels have been set, cannot be stimulated further to lower AMPA receptors in membranes. **(v)** The reduced explorative behavior (Figure 6D), enhanced stereotypic self-grooming (Figure 6E), and motor activity (Figure 5E), as well as the elevated corticosterone levels (Figure 6I) of *Bdnf^*Pax*2^*KOs, reveal deficits in social learning and increased anxiety that may occur as a result of impaired stress control and novelty discrimination. Both stress control and novelty discrimination require proper AMPA receptor trafficking (Derkach et al., 2007; Blair et al., 2019; Penrod et al., 2019; Roth et al., 2020). **(vi)** Finally, various phenotypic characteristics of *Bdnf^*Pax*2^*KO mice are reminiscent of mouse models relevant to neurodevelopmental disorders, such as ASD. These include reduced PV-IN labeling (Takano, 2015; Pirone et al., 2018; Goel et al., 2019), elevation of Arc levels (Korb and Finkbeiner, 2011; Goel et al., 2019), increased fEPSPs (Mohn et al., 2014), as well as elevated corticosterone levels (Das et al., 2019). Also, a mouse line deficient in adenomatous polyposis coli protein, a key regulator of synapse maturation (Hickman et al., 2015), also developed an autistic phenotype (Mohn et al., 2014; Alexander et al., 2020). These mice showed a reduced dynamic range of hearing and deficits in IHC synapses linked to altered high-SR and low threshold characteristics (Hickman et al., 2015), features similar to those observed in *Bdnf^*Pax*2^*KO mice.

Fast inhibitory PV-INs are known to be important for gamma- (feed-forward inhibition) and beta-oscillations (feedback inhibition) (Sohal et al., 2009). A diminished activity in tonic fast-spiking PV-IN networks in rodent animal models for ASD was linked to enhanced baseline spontaneous gamma-band power and reduced beta oscillations (Gill and Grace, 2014). Interestingly, in children with fast auditory processing deficits and ASD, elevated and spontaneous baseline gamma-band power was recently found to be linked to reduced evoked gamma power (Foss-Feig et al., 2017; Mamashli et al., 2017; McCullagh et al., 2020).

**In Conclusion:** We propose that BDNF in GABAergic IN precursors contributes to the shaping of the tonic inhibitory conductance of hindbrain neurons through sensory experience. As shown here for auditory DCN brainstem neurons, tonic inhibitory strength is reduced in *Bdnf^*Pax*2^*KOs and linked to elevated SFR and thresholds. Proper tonic inhibitory shaping is required to decrease the membrane time constant of sensory neurons in order to narrow the temporal window for synaptic integration by affecting background spontaneous firing rates (Kopp-Scheinpflug et al., 2011). This ensures a high signal-to-noise ratio for the transfer of specific, sensory-evoked information and filters signals that are not associated with the sensory stimuli, as previously also shown for sound-induced brainstem responses (Kopp-Scheinpflug et al., 2011). The findings in the present study may indicate that BDNF in Pax2-lineage descendent cells influences fast auditory processing. Fast auditory processing deficits following for example early brainstem injuries in children have been associated with cognitive deficits, including the failure to properly process rapidly changing acoustic information, a prerequisite during the acquisition of language and social learning (Fitch et al., 1997; Fitch and Tallal, 2003; Ramus, 2003; Rendall et al., 2017). Heterozygous Pax2 mice (Wei et al., 2020), exhibited an autistic-like pattern, evidenced through increased self-grooming and anxiety, although normal social behavior and working memory (Wei et al., 2020). It may be interesting to consider defects in BDNF expression in Pax2-deficient cells in future studies.

Numerous studies that predicted that abnormalities in the migration of GABAergic INs from subpallium areas to the cortex are a key factor underlying etiologies of various neurodevelopmental disorders including ASD, epilepsy, schizophrenia, anxiety, and depression (Levitt et al., 2004; Lewis et al., 2005; Marin, 2012; Kiss et al., 2014; Southwell et al., 2014; Takano, 2015; Shetty and Bates, 2016; Su et al., 2016; Reim and Schmeisser, 2017; Rendall et al., 2017; Li et al., 2018; Malhi and Mann, 2018; Peng et al., 2018), may now consider that, in addition, defects in targeting of GABAergic INs to lower hindbrain regions may contribute to neurodevelopmental problems, such as ASD.

## Materials and Methods

### Animals

*Bdnf^*Pax*2^*KO and control mice were obtained by crossing a Cre line, in which Cre is expressed under the promoter of the *Pax2* gene and a mouse line in which the protein coding *Bdnf-exon IX* is flanked by *loxP* sites. Both lines were obtained from the Mutant Mouse Regional Research Center, MMRRC (Rios et al., 2001; Ohyama and Groves, 2004; Zuccotti et al., 2012). To verify the deletion pattern of *Bdnf*, *Pax2-*Cre mice were crossed with Rosa^tdTomato^ reporter mice (Madisen et al., 2010). Deletion of the *Bdnf* gene in distinct brain areas of *Bdnf^*Pax*2^*KO was verified by Northern and Western blots. Genotyping of the mouse lines was performed as described (Rios et al., 2001). For all experiments, mice of either sex were used. The ages of adult animals were between 2 and 6 months, while for juveniles, the age is given in the respective results section. The sample size was chosen with the experience of previous publications, recommendations in literature, and on the basis of the expected effect size *n* calculated with G power. The care and use of mice and the experimental protocol were reviewed and approved by the University of Tübingen, Veterinary Care Unit, and by the Animal Care and Ethics Committee of the Regional Board of the Federal State Government of Baden-Württemberg, Germany, and followed the guidelines of the European Union Directive 2010/63/EU for animal experiments.

### Co-localization of mRNA and Protein in Brain Sections

Animals were deeply anesthetized with CO_2_ and then sacrificed by decapitation. Brain tissue was prepared and sectioned with a vibratome at 60 μm, as previously described (Singer et al., 2016). mRNA and protein were co-localized on free-floating brain sections as previously described (Singer et al., 2014). In brief, following prehybridization for 1 h at 37°C, sections were incubated overnight with BDNF or Arc riboprobes at 56°C, incubated with anti-digoxigenin antibody conjugated to alkaline phosphatase (anti-Dig-AP, Roche, Germany, 11093274910), and developed as previously described (Singer et al., 2013). For protein detection, streptavidin–biotin was blocked according to the manufacturer’s instructions (Streptavidin–Biotin Blocking Kit, Vector Laboratories, United States) after blocking endogenous peroxidase. Sections were incubated overnight at 4°C with the primary antibodies against Arc/Arg3.1 (Synaptic Systems, Germany, anti-rabbit, 1:200, 156003) (Nikolaienko et al., 2018) or parvalbumin (Abcam, United Kingdom, anti-rabbit, 1:500, ab11427), followed by incubation with the secondary antibody (biotinylated goat anti-rabbit, Vector Laboratories, BA-1000) and chromogenic detection (AEC, 3-amino-9-ethylcarbazole, Vector Laboratories, SK-4200). For co-labeling of BDNF mRNA and tdTomato, brain slices of Pax2-CRE-Rosa^tdTomato^ reporter mice were taken. For BX61 microscopy (Olympus, Japan) evaluation photographs were taken with a florescence camera (XM 10, Olympus, Japan) for detection of tdTomato florescence, and with a bright-field camera (DP 71, Olympus, Japan) for detection of mRNA and protein, without adjusting the picture frame or the plane of focus. As confocal microscopy is not possible, the resolution at higher magnification was limited.

### Immunohistochemistry

Animals were deeply anesthetized with CO_2_ and then sacrificed by decapitation. Brain tissue for fluorescence-immunohistochemistry was prepared and sectioned with a vibratome at 60 μm, as previously described (Singer et al., 2016). The sections of mouse brains were stained as described (Tan et al., 2007; Singer et al., 2016). Antibodies directed against parvalbumin (Abcam, United Kingdom, anti-rabbit, 1:2000, ab11427) and vGluT2 (Synaptic Systems, Germany, anti-mouse, 1:500, 135421) were detected using appropriate Alexa 488 (Molecular Probes, Germany, 1:500, A11001) and Cy3 (Jackson Immuno Research Europe, United Kingdom, 1:500, 711-166-152) conjugated secondary antibodies. Sections were viewed using a BX61 microscope (Olympus, Japan), as previously described (Zampini et al., 2010).

### Field Excitatory Postsynaptic Potential (fEPSP) Recordings in Hippocampal Slices

Animals were deeply anesthetized with CO_2_ and then sacrificed by decapitation. Extracellular fEPSP recordings were performed according to standard methods, as previously described (Matt et al., 2011; Ngodup et al., 2015; Chenaux et al., 2016). In brief, stimulation (TM53CCINS, WPI) and recording (ACSF-filled glass pipettes, 2–3 MΩ) electrodes were positioned in the stratum radiatum (SR) to record Schaffer collateral field excitatory postsynaptic potentials (fEPSPs). The same stimulus intensity was applied during baseline recording (0.067 Hz, 20–30 min) and induction of LTP using 100 Hz stimulation for 1 sec or LTD using 1 Hz stimulation for 15 min. The baseline was determined by averaging fEPSP initial slopes from the period before the LTP or LTD stimulation. The level of LTP/LTD was determined by averaging fEPSP slopes from the period between 50 and 60 min after the high-frequency/low-frequency stimulation. Before the LTP/LTD stimulation, each slice was used to record input-output relationship (25–150 μA in 25 μA steps) and paired-pulse facilitation (10–20–50–100–200–500 ms interpulse interval at the same stimulation strength as LTP/LTD recordings). For IOR changes in fEPSP slope were averaged for each group and plotted against the stimulus intensity. For PPF paired-pulse ratio of EPSP2/EPSP1 slope at each interstimulus interval were defined per slice and mean values per group were plotted. EPSP1 was calculated as an average of EPSP1s from all interstimulus intervals for each single slice. Four traces were averaged (WinWCP V5.5.3) for each single analyzed data point.

### Multiple T-Maze

The maze consisted of nine equally sized T-elements (8 × 4.5 × 0.4 cm, Lange-Asschenfeldt et al., 2007) made of PVC. The maze also included a start element (14 × 4.5 cm) and a target platform (19 × 12.6 cm). Each element was mounted on a stand; the total element height was 23 cm. To reach the target platform 7 decision points needed to be passed (order: LRRLLRL). The individual mouse house (Tecniplast, Italy) from the home cage was placed on the target platform. Mice were trained on the maze for three consecutive days. On day 1 and 2, each mouse had three runs. If a mouse reached the target platform within the time limit of 10 min, it was scored as a successful run; if not, the trial was terminated. On day 3, each mouse had to perform as many runs as necessary to reach 7 successful training runs. Mice were re-tested twice after a break of 3 and 18 days following their last training run. The sequence in which the mice were placed on the maze was pseudorandomized and then maintained throughout the experiment. Experiments were performed between 10 am and 6 pm. After each run, the maze was cleaned with 70% ethanol. The average light intensity in the maze was 75 lux.

### Motor Activity

The force of the mouse movement was measured during acoustic startle experiments (not shown). Motor activity was measured in the 50 ms time window before the stimulus was presented with a piezoelectric force transducer situated inside a sound-attenuated chamber by calculation of the peak-to-peak force. The apparatus consisted of a measuring platform with a wire mesh test cage with a metal floor plate (5 × 9 × 5 cm). The output of the transducer was amplified and filtered from 2 to 150 Hz (University of Tuebingen, Piezo-Amp-System, Tuebingen, Germany). The resulting voltage was sampled (1 kHz) by an analog-to-digital converter located within a computer (Microstar DAP 1200, Washington, DC), results are given in mN (milli-Newton).

### Social-Interaction Test

The apparatus for Crawley’s sociability test (Silverman et al., 2010) consisted of a rectangular three-chamber PVC box in which each compartment had an area of 19 × 45 cm. In the outer chambers, two identical wired cup-like containers were placed. In one of them, a “stranger” (mouse of the same background, age and gender but without prior contact to the subject mouse) was placed. In the other chamber, an empty container worked as a novel object. The experimental mouse was placed in the center compartment for 5 minutes to adapt, while the lateral compartments were isolated by dividing walls. The walls were then removed and the experimental mouse was allowed to discover all three chambers for 10 minutes. The behavioral testing was performed between 9 am and 5 pm. After each trial, the chambers were cleaned with 70% ethanol.

### Ultrasonic Vocalization

Ultrasonic vocalizations of P7 pups were recorded to analyze the reaction of short (5 min) separation from the parental cage as described in Kromer et al. (2005) and Groenink et al. (2008). The pups were randomly selected, the body weight was measured and the single animal was placed on a fresh paper towel in the middle of a plastic box (13 × 13 cm) in a soundproofed chamber with constant temperature of 23 ± 1°C. An ultrasonic microphone (Neutrix), connected with a preamplifier (Avisoft UltraSoundGate416) was fixed with a distance of 12 cm from the middle of the experimental box. For recording, Avisoft (Avisoft Bioacoustic RECORDER Version 4.2.29) was used with a sampling rate of 250 kHz which allows a frequency range from 0 to 125 kHz. With SASlab (Avisoft-SASLabPro Version 5.2.13) a spectrogram of the recordings was calculated and the number of calls was counted manually.

### Blood Corticosterone Level Analysis

Blood was collected from the tail vein of anesthetized mice (anesthesia see section *Hearing Measurements and Sound Exposure*) within 5 min after injection, centrifuged, and stored at –80°C. The corticosterone concentration in the blood was measured using a Corticosterone ELISA Kit (Enzo Life Sciences, Farmingdale, NY, United States). The optical density of the samples was finally read at 405 nm in the FLUOstar Optima (BMG LABTECH GmbH, Ortenberg, Version 2.20). To rule out major influences of the circadian rhythm, all blood was taken in the afternoon (between 3 and 5 pm).

### *In vivo* Recordings in the Dorsal Cochlear Nucleus (DCN)

Juxtacellular single-unit recordings were performed in adult controls and in *Bdnf^*Pax*2^*KOs. The experimental protocol for such recordings was described previously (Müller et al., 2019). Animals were intraperitoneally anesthetized with a mixture of ketamine hydrochloride (0.1 mg/g bodyweight; Ketamin-Ratiopharm, Ratiopharm) and xylazine hydrochloride (5 μg/g bodyweight; Rompun, Bayer). Throughout recording sessions, anesthesia was maintained by additional subcutaneous application of one-third of the initial dose approximately every 60 min. Briefly, recordings were performed in a sound-attenuating chamber (Type 400, Industrial Acoustic Company) with the animal stabilized in a custom-made stereotaxic apparatus. Acoustic stimuli were digitally generated using custom-written Matlab functions (version 7.5, The MathWorks Inc, Natick, United States, RRID:SCR_001622). The stimuli were transferred to a D/A converter (RP2.1 real-time processor, 97.7 kHz sampling rate, Tucker-Davis Technologies) and delivered through custom-made earphones (acoustic transducer: DT 770 pro, Beyer Dynamics). Juxtacellular recordings of DCN single-units were performed with glass micropipettes (GB150F-10, Science Products, 5-10 MΩ) filled with 3M KCl. The DCN was approached dorsally, and reached at penetrations depths of 3500-4000 μm. Fusiform cells were identified based on the biphasic waveform, V-shaped FRA, and pauser/build-up PSTH (Rhode et al., 1983; Rhode and Smith, 1986; Felix et al., 2017). Subsequently, the mouse was perfused transcardially with 0.9% NaCl solution followed by 5% PFA. Coronal slices containing the cochlear nucleus were cut on a vibratome (HM 650V, Microm), and the tissue sections were visualized under a fluorescent microscope (Zeiss Axioskop 2). The recording sites were histologically verified by iontophoretic injection of Flurogold (+5 μA, 5 min).

### Hearing Measurements and Sound Exposure

The hearing function of adult *Bdnf^*Pax*2^*KO and controls was studied by measuring and analyzing auditory brainstem responses (**ABR**) and auditory steady-state responses (**ASSR**), as previously described (Zuccotti et al., 2012; Rüttiger et al., 2013; Wolter et al., 2018). Animals were exposed to enriching sound as described (Matt et al., 2018). Animals were anesthetized with intraperitoneal injections of fentanyl (0.05 mg/kg bodyweight, Fentadon; Albrecht GmbH, Aulendorf, Germany), midazolam (2.5 mg/kg body weight, Midazolam-hameln; Hameln Pharma plus GmbH, Hameln, Germany), medetomidin (0.5 mg/kg bodyweight, Sedator; Albrecht GmbH, Aulendorf, Germany) and atropine sulfate (0.2 mg/kg body weight, B. Braun, Melsungen, Germany). Additional doses of anesthetics were administered if needed.

### Quantification and Statistical Analysis

#### Statistics and Numbers

All statistical information, including the statistical tests and *post hoc* tests used, the exact value of *n*, what n represents and the precision documentation of statistical outcome, can be found in Supplementary Table 1. Basic statistical information, such as *P-*values and *n*, can be found in the figure legends. In the figures, significance is indicated by asterisks (^^*^P < 0.05, ^∗∗^*P* < 0.01, ^∗∗∗^*P* < 0.001, and ^****^P < 0.0001). n.s. denotes non-significant results (*P* > 0.05). A trend is indicated by asterisk in brackets [(^∗^) *P* < 0.1].

#### Colocalization of mRNA and Protein and Immunohistochemistry in Brain Sections

Brain sections were quantified by integrating density values of color pixels for each single specimen using ImageJ software. The density values of all specimens stained within the same experiment were then normalized to the group mean (i.e., all hippocampal brain sections stained in the same experiment gave an average value of 1.0) or, in the case of development studies, they were normalizedto all animals in the age range of P6 to P10. This correction allowed compensating for the high inter-trial variation of staining intensity. All sections from one mouse were then averaged and entered the statistical evaluation as *n* = 1.

#### fEPSP Recordings in Hippocampal Slices

Data was processed and analyzed using WinWCP V5.5.3, Clampfit 10.7 (Molecular Devices), Microsoft Excel and GraphPad Prism 8. The data presented per experimental group/condition contained (additionally to mean ± SEM) single dots which showed the fEPSP slope values for each individual brain slice. The *n* indicates the number of slices and animals (slices/animals) used in the analysis.

#### Multiple T-Maze

Each trial was video-recorded with a webcam (Logitech c920). If a mouse fell from the maze, it was immediately placed back on the same spot. Errors were counted offline using the software BORIS (Friard and Gamba, 2016). An error was counted when a mouse deviated from the correct path to the target with all four paws. Consecutive errors made at the same decision point were counted as one error. Statistics was calculated with JMP 14 (SAS Institute Inc., United States). The circling behavior was measured by counting the number of full 360° rotations during the time in the maze. Data from two mice, which failed to find the target platform during the first 5 training runs, were excluded from further analysis. Time measurement was stopped when a mouse reached the mouse house with all four paws.

#### Social-Interaction Test

The duration of sniffing contact of the mouse at the stranger- and the empty container were normalized to the time spent in the respective compartment, and the number of entries the mouse made in each of the compartments, the latency to the first entry into each chamber, as well as the time the experimental mouse spent with freezing or grooming during the 10 min period was analyzed. The circling behavior was measured by counting the number of full 360° rotations during the habituation time.

#### Blood Corticosterone Level Analysis

The values measured for optical density were exported to Excel (Microsoft, 2016) and analyzed according to manufacturer’s instructions found online at myassays.com.

#### *In vivo* Recordings in the Dorsal Cochlear Nucleus (DCN)

Response threshold (the lowest stimulus level resulting in an increase of spiking), characteristic frequency (**CF**, the sound frequency causing increased firing at the lowest sound level), and maximum discharge rate were analyzed as described (Müller et al., 2019). The quality factor (Q_10_) was calculated as the ratio between the unit’s CF and the frequency bandwidth (CF/BW) at 10 dB above threshold. The dB range between 10 and 90% of the rising slope of the rate-level function at CF was defined as the dynamic range. For units with prominent inhibitory sidebands, indicated by a significant decrease in firing below the spontaneous rate, inhibitory strength was calculated as the relative reduction of the firing rate within the inhibitory sideband with respect to the rate outside of excitatory receptive field (“non-inhibitory area”) (Chumak et al., 2016). In addition, the ratio between AP discharge rates in excitatory and non-inhibitory areas was calculated (Chumak et al., 2016). Peri-stimulus time histograms were used to determine the first spike latency (**FSL**), calculated as the time between stimulus onset and the peak of a kernel density function (Botev et al., 2010) fitted over the AP spike times.

#### ABR Wave Form Analysis

Auditory brainstem responses waveforms were analyzed for consecutive amplitude deflections (waves), with each wave consisting of a starting negative (n) peak and the following positive (p) peak. Wave latencies were defined by the onset timing (negative peak) of each corresponding wave. Peak amplitudes and latencies of ABR waves I and IV were extracted and defined as wave I: I_n_ - I_p_ (0.85-1.9 ms); wave IV: IV_n_ − IV_p_ (3.15-6.05 ms). A customized computer program (Peak, University of Tübingen) was used to extract ABR peak amplitudes and latencies based on these definitions. From the extracted peaks, ABR peak-to-peak (wave) amplitude and latency growth functions (Burkard and Don, 2007) were calculated for individual ears for increasing stimulus levels. All ABR wave amplitude and latency growth functions were normalized with reference to the ABR thresholds (from -10 dB to a maximum of 90 dB relative to threshold for wave amplitudes and from 0 dB to a maximum of 90 dB above threshold for wave latencies).

## Significance Statement

The present findings demonstrate a requirement for BDNF in Pax2-lineage descendants (GABAergic precursors) in hindbrain regions for the development of proper cognitive abilities. *Bdnf*^*Pax2*^KO mice lack proper maturation of fine-grained resolution of auditory brainstem output activity, maturation of dendritic outgrowth of PV–INs and scaled Arc levels in the auditory cortex and hippocampus, required for LTP/LTD adjustments, learning, and control of anxiety and social behavior. BDNF in Pax2-lineage descendants in lower brainstem regions may thus be involved in the disturbed migration of GABAergic INs which may contribute to the pathophysiology of multiple psychiatric disorders, including autism.

## Data Availability Statement

The raw data supporting the conclusions of this article will be made available by the authors, without undue reservation.

## Ethics Statement

The animal study was reviewed and approved by the Animal Care and Ethics Committee of the Regional Board of the Federal State Government of Baden-Württemberg, Germany.

## Author Contributions

PE, PM, LR, and MK designed the research. PE, PM, MM, MW, SJ, DS, LR, IM, PP, and MK performed the research. PE, PM, MM, MW, SJ, DS, WS, LR, and PP contributed to unpublished reagents and analytic tools and analyzed the data. PE and MK wrote the first draft of the manuscript. MJ, TS, PP, and MK edited the manuscript. PE, PM, LR, and MK wrote the manuscript. All authors contributed to the article and approved the submitted version.

## Conflict of Interest

The authors declare that the research was conducted in the absence of any commercial or financial relationships that could be construed as a potential conflict of interest.

## Supplementary Material

Supplementary Figure 1 Hippocampal fEPSP slope in *Bdnf*^*Pax2*^KOs. **(A)** Averaged fEPSP slope was plotted as a function of stimulus intensity. No difference was observed between slices from controls, *Bdnf*^*Pax2*^KOs, P6-10 controls and enriching sound-exposed (SE) controls and *Bdnf*^*Pax2*^KOs. Traces from representative recordings are shown on the right (control: *n* = 9/14 animals/slices, *Bdnf*^*Pax2*^KOs: *n* = 9/10 animals/slices, P6-10 controls *n* = 6/7 animals/slices, controls exposed *n* = 4/11 animals/slices, *Bdnf*^*Pax2*^KOs exposed *n* = 3/5 animals/slices; *P* = 0.54). **(B)** Paired pulse facilitation was not different between slices from controls, *Bdnf*^*Pax2*^KOs, P6-10 controls and sound-exposed controls and *Bdnf*^*Pax2*^KOs for all inter-stimulus intervals. Traces from representative recordings are shown on the right (controls: *n* = 9/14 animals/slices, *Bdnf*^*Pax2*^KOs: *n* = 9/10 animals/slices, P6-10 controls *n* = 6/7 animals/slices, controls exposed *n* = 4/11 animals/slices, *Bdnf*^*Pax2*^KOs exposed *n* = 3/5 animals/slices; *P* = 0.75). Mean ± S.E.M.

Supplementary Figure 2 Hearing function of *Bdnf*^*Pax2*^KOs. **(A)** Click evoked (*n* = 23-26/46-52 mice/ears; *P* = 0.002), noise burst (*P* = 0.026) and frequency-specific ABR thresholds (*n* = 16/32 mice/ears each; *P* < 0.0001). Schematic ABR waveform of controls (black) and *Bdnf*^*Pax2*^KOs (red) in relation to the corresponding auditory nuclei in the ascending auditory pathway. **(B)** Noise stimulus-evoked amplitude of AN activity (left panel) and latency (right panel) (amplitude: *n* = 12-14/24-28 mice/ears; *P* < 0.0001; latency: *n* = 12-14/24-28 mice/ears; *P* < 0.0001). **(C)** Noise stimulus-evoked amplitude of midbrain activity (left panel) and latency (right panel) (Amplitude: *n* = 12-14/24-28 mice/ears; *P* < 0.0001; latency: *n* = 12-14/24-28 mice/ears; *P* < 0.0001). **(D)** The signal to noise ratio (SNR) of modulation depth response (left panel; *n* = 10/10 mice/ears each; *P* = 0.001) and SNR modulation I-O function of an amplitude-modulated tone (right panel; *n* = 10/10 mice/ears each; *P* < 0.0001) was reduced in *Bdnf*^*Pax2*^KOs. Mean ± S.E.M.

Supplementary Table 1 Detailed statistical information.
