# Supplementary figures and images for: Deletion of BDNF in Pax2 Lineage-Derived Interneuron Precursors in the Hindbrain Hampers the Proportion of Excitation/Inhibition, Learning, and Behavior
Source: Front Mol Neurosci. 2021 Mar 26;14:642679. doi: 10.3389/fnmol.2021.642679 (PMC8033028; doi:10.3389/fnmol.2021.642679)

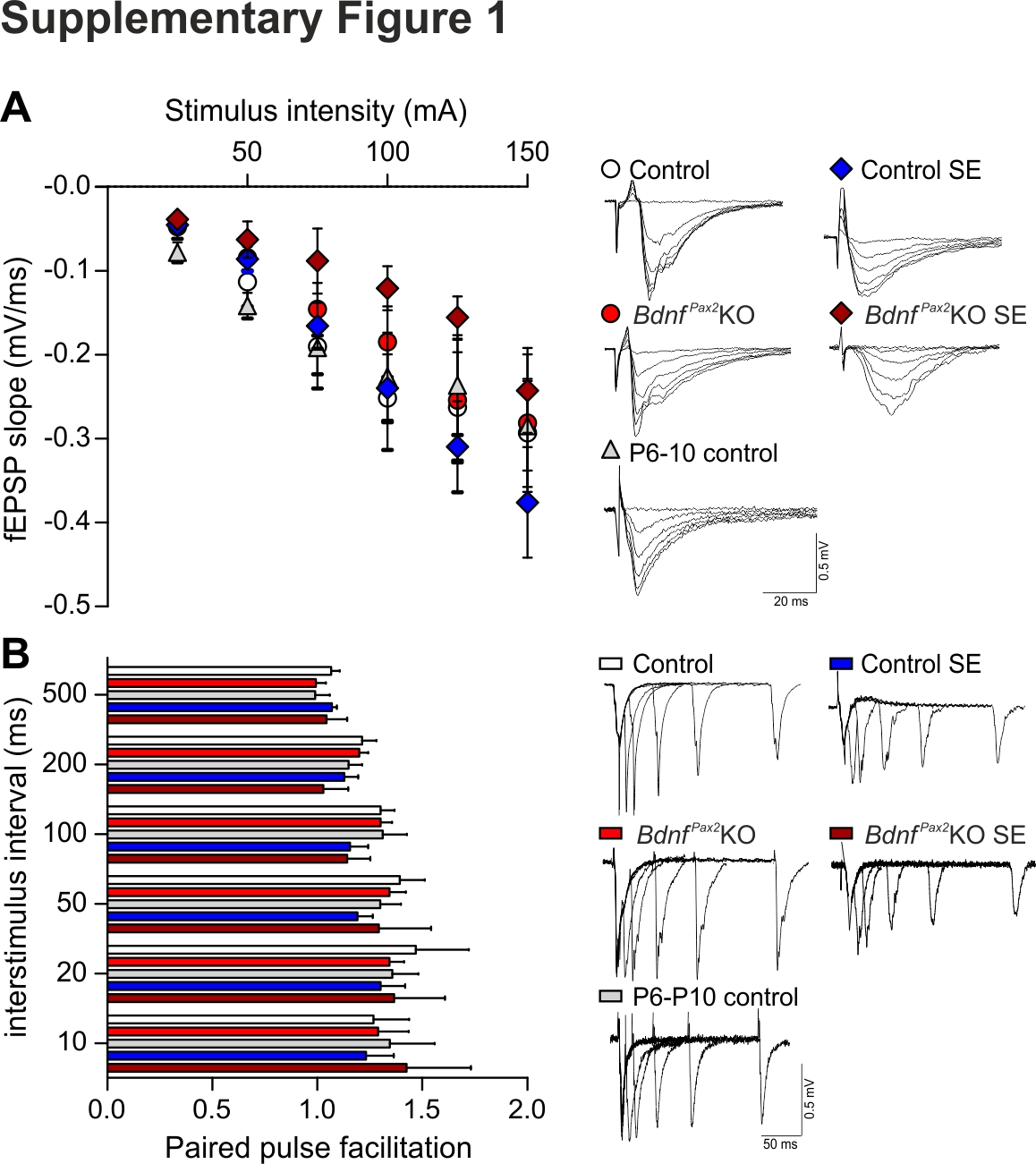

Supplement: Supplementary Figure 1 — Hippocampal fEPSP slope in BdnfPax2KOs. (A) Averaged fEPSP slope was plotted as a function of stimulus intensity. No difference was observed between slices from controls, BdnfPax2KOs, P6-10 controls and enriching sound-exposed (SE) controls and BdnfPax2KOs. Traces from representative recordings are shown on the right (control: n = 9/14 animals/slices, BdnfPax2KOs: n = 9/10 animals/slices, P6-10 controls n = 6/7 animals/slices, controls exposed n = 4/11 animals/slices, BdnfPax2KOs exposed n = 3/5 animals/slices; P = 0.54). (B) Paired pulse facilitation was not different between slices from controls, BdnfPax2KOs, P6-10 controls and sound-exposed controls and BdnfPax2KOs for all inter-stimulus intervals. Traces from representative recordings are shown on the right (controls: n = 9/14 animals/slices, BdnfPax2KOs: n = 9/10 animals/slices, P6-10 controls n = 6/7 animals/slices, controls exposed n = 4/11 animals/slices, BdnfPax2KOs exposed n = 3/5 animals/slices; P = 0.75). Mean ± S.E.M. [file Image_1.jpg]

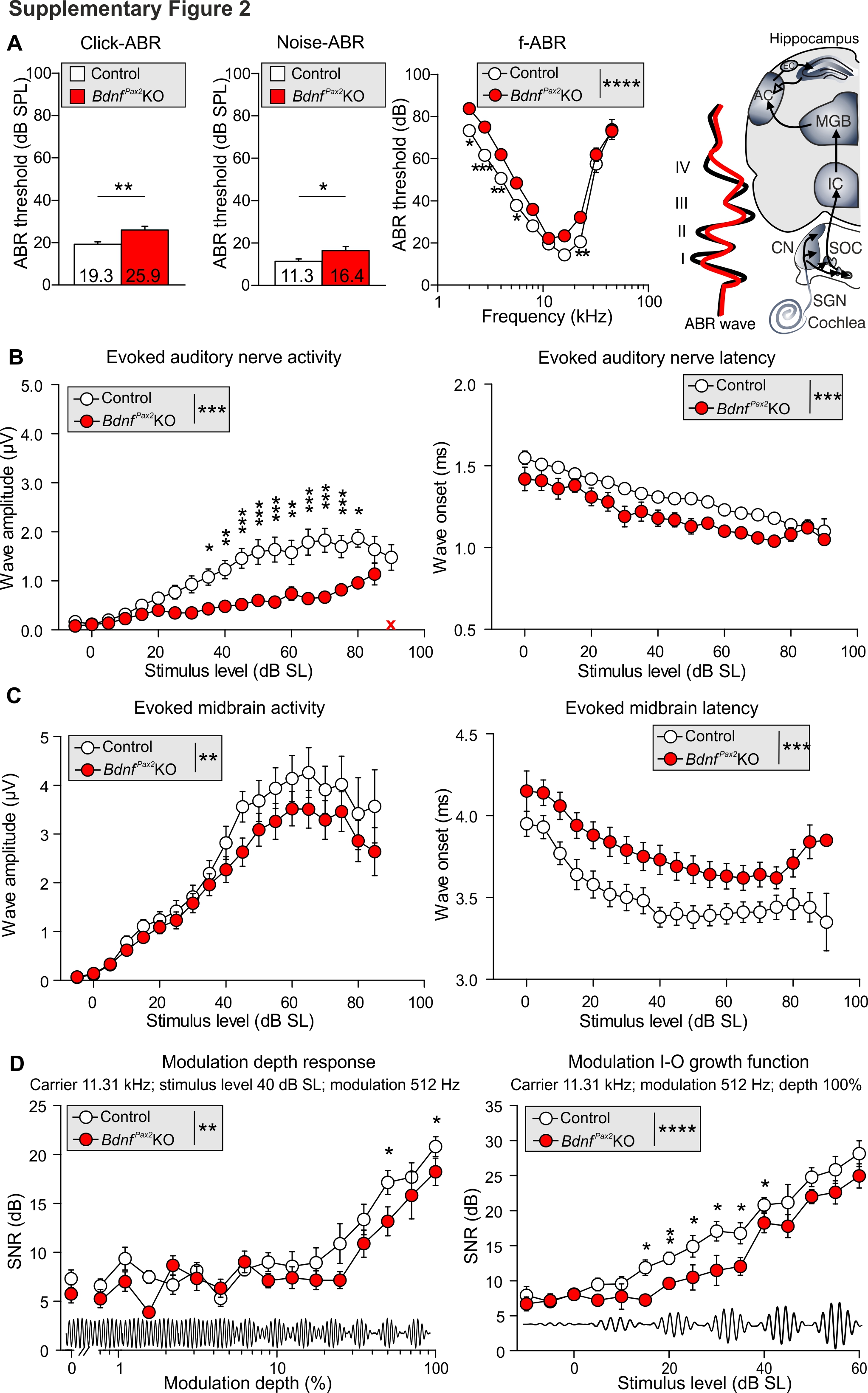

Supplement: Supplementary Figure 2 — Hearing function of BdnfPax2KOs. (A) Click evoked (n = 23-26/46-52 mice/ears; P = 0.002), noise burst (P = 0.026) and frequency-specific ABR thresholds (n = 16/32 mice/ears each; P < 0.0001). Schematic ABR waveform of controls (black) and BdnfPax2KOs (red) in relation to the corresponding auditory nuclei in the ascending auditory pathway. (B) Noise stimulus-evoked amplitude of AN activity (left panel) and latency (right panel) (amplitude: n = 12-14/24-28 mice/ears; P < 0.0001; latency: n = 12-14/24-28 mice/ears; P < 0.0001). (C) Noise stimulus-evoked amplitude of midbrain activity (left panel) and latency (right panel) (Amplitude: n = 12-14/24-28 mice/ears; P < 0.0001; latency: n = 12-14/24-28 mice/ears; P < 0.0001). (D) The signal to noise ratio (SNR) of modulation depth response (left panel; n = 10/10 mice/ears each; P = 0.001) and SNR modulation I-O function of an amplitude-modulated tone (right panel; n = 10/10 mice/ears each; P < 0.0001) was reduced in BdnfPax2KOs. Mean ± S.E.M. [file Image_2.jpg]
